# CD8 positive T-cells decrease neurogenesis and induce anxiety-like behaviour following hepatitis B vaccination

**DOI:** 10.1093/braincomms/fcae315

**Published:** 2024-09-16

**Authors:** Tuo Zhou, Yuxuan Gao, Zhiling Wang, Chunfang Dai, Ming Lei, Aubrey Liew, Sen Yan, Zhibin Yao, Dandan Hu, Fangfang Qi

**Affiliations:** Children's Health Section, Guangzhou Women and Children's Medical Center, Guangzhou Medical University, Guangzhou 510623, China; Breast Disease Center, The First Affiliated Hospital of Sun Yat-Sen University, Sun Yat-Sen University, Guangzhou 510080, China; Department of Orthopedic Surgery, Sun Yat-Sen Memorial Hospital, Sun Yat-Sen University, Guangzhou 510120, China; Children's Health Section, Guangzhou Women and Children's Medical Center, Guangzhou Medical University, Guangzhou 510623, China; Department of Neurology, Sun Yat-Sen Memorial Hospital, Sun Yat-Sen University, Guangzhou 510120, China; Department of Immunology, Mayo Clinic, Rochester 55905, USA; Guangdong Key Laboratory of Non-human Primate Research, Guangdong-Hongkong-Macau Institute of CNS Regeneration, Jinan University, Guangzhou 519070, China; Department of Anatomy and Physiology, Zhongshan School of Medicine, Sun Yat-Sen University, Guangzhou 510080, China; Children's Health Section, Guangzhou Women and Children's Medical Center, Guangzhou Medical University, Guangzhou 510623, China; Department of Anatomy and Physiology, Zhongshan School of Medicine, Sun Yat-Sen University, Guangzhou 510080, China; Department of Neurology, Mayo Clinic, Rochester 55905, USA

**Keywords:** hepatitis B vaccine, neurogenesis, TNF-α, CXCL16, CXCR6

## Abstract

Mounting evidence indicates the involvement of peripheral immunity in the regulation of brain function, influencing aspects such as neuronal development, emotion, and cognitive abilities. Previous studies from our laboratory have revealed that neonatal hepatitis B vaccination can downregulate hippocampal neurogenesis, synaptic plasticity and spatial learning memory. In the current post-epidemic era characterized by universal vaccination, understanding the impact of acquired immunity on neuronal function and neuropsychiatric disorders, along with exploring potential underlying mechanisms, becomes imperative. We employed hepatitis B vaccine-induced CD3 positive T cells in immunodeficient mice to investigate the key mechanisms through which T cell subsets modulate hippocampal neurogenesis and anxiety-like behaviours. Our data revealed that mice receiving hepatitis B vaccine-induced T cells exhibited heightened anxiety and decreased hippocampal cell proliferation compared to those receiving phosphate-buffered saline-T cells or wild-type mice. Importantly, these changes were predominantly mediated by infiltrated CD8^+^ T cells into the brain, rather than CD4^+^ T cells. Transcriptome profiling of CD8^+^ T cells unveiled that C-X-C motif chemokine receptor 6 positive (CXCR6^+^) CD8^+^ T cells were recruited into the brain through microglial and astrocyte-derived C-X-C motif chemokine ligand 16 (CXCL16). This recruitment process impaired neurogenesis and induced anxiety-like behaviour via tumour necrosis factor-α-dependent mechanisms. Our findings highlight the role of glial cell derived CXCL16 in mediating the recruitment of CXCR6^+^CD8^+^ T cell subsets into the brain. This mechanism represents a potential avenue for modulating hippocampal neurogenesis and emotion-related behaviours after hepatitis B vaccination.

## Teaser

Lui and Venkatraman report that normalized hippocampal sodium MRI signal were more predictive of Alzheimer's disease assessment scale cognitive subscale and mini-mental state examination scores in a non-demented older adult cohort than hippocampal cortical volumes and conclude that sodium MRI may have applications as an imaging neurocognitive marker.

## Graphical Abstract

**Graphical Abstract:**
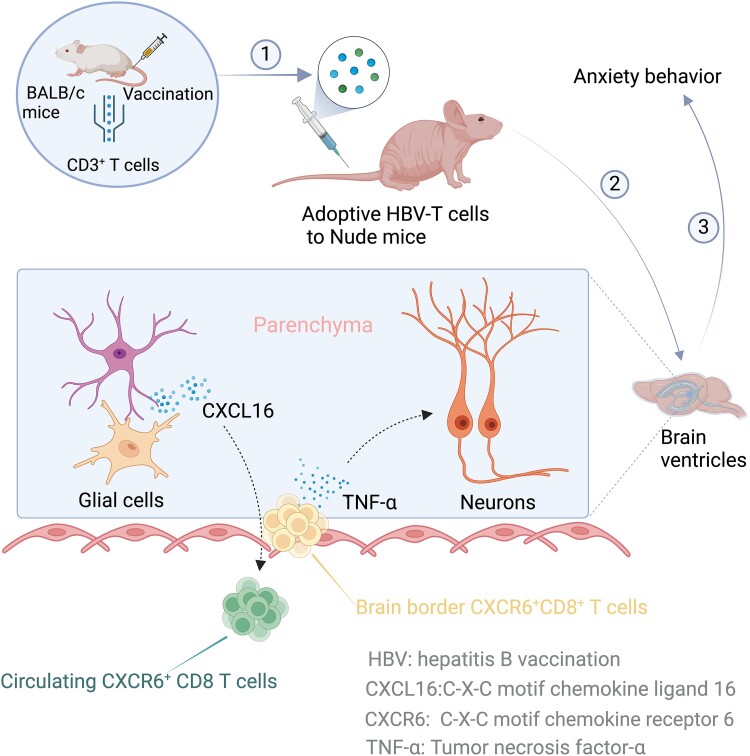

## Introduction

The adaptive immune system plays pivotal roles in diverse aspects of brain function, encompassing brain development, neurogenesis, white matter aging, and the onset of neuropsychiatric disease.^1^ Previous investigations from our laboratory have unveiled that the activation of the adaptive immune system triggered by the hepatitis B vaccine results in a transient reduction in hippocampal neurogenesis, synaptic plasticity and spatial learning abilities. These effects were tentatively associated with CD4^+^ T helper (Th) 2 bias and the diverse activation phenotypes of microglia. However, emerging evidence suggests that CD8^+^ T cells, representing another crucial subset within adaptive immunity, may also infiltrate the brain and contribute to diminished neurogenesis and cognitive decline.^2^ Conversely, disparate studies indicate that brain resident CXCR6^+^CD8^+^ T cells can mitigate Alzheimer's disease (AD) pathology, foster hippocampal neurogenesis and influence behaviour.^3,4^ Furthermore, investigations have illuminated the role of CXCR6 in conjunction with CXCL16 signalling, regulating the recruitment of CD8^+^ T cells in the contexts of healthy aging, cognitive impairment and AD.^2,3,5^

With ∼85% of neonates worldwide undergoing the three-dose hepatitis B vaccine regimen for viral infection prevention,^6^ it becomes imperative to unravel the impact of adaptive immune activation, particularly post-hepatitis B vaccination (HBV), on mood states and hippocampal neurogenesis. Hence, our hypothesis posits that the CD8^+^ T cell subset may serve as a mediator in the observed reduction in neurogenesis and the induction of anxiety-like behaviour following HBV vaccination. Our subsequent investigation aims to elucidate the mechanisms underlying the recruitment of CD8^+^ T cells into the brain and how their activation contributes to changes in neurogenesis and mood states. Combining isolated CD8^+^ T cell RNA sequencing and flow cytometry, we provide compelling evidence that the HBV-induced decline in hippocampal neurogenesis and the manifestation of anxiety-like behaviour are mediated by infiltrating CD8^+^ T cells through TNF-α-dependent mechanisms.

## Materials and methods

### Animals and housing conditions

The experimental procedures were conducted in strict adherence to ethical guidelines and were approved by the Animal Care and Use Committee of Sun Yat-Sen University. All protocols conformed to the Guide for the Care and Use of Laboratory Animals as stipulated by the National Institutes of Health, USA. Male BALB/c mice (6 weeks) serving as donors of T cells, and male immunodeficient nude mice (6 weeks), utilized as recipients of T cells, were purchased from the Sun Yat-Sen University Laboratory Animal Centre in Guangzhou, China. The mice were housed in same-sex pairs within a specific pathogen-free facility. The colony was meticulously maintained on 12-h light/dark cycles, with continuous access to food and water, all within a temperature- and humidity-controlled environment. The sample size was chosen based on similar previous studies.^7,8^ All mice were randomly assigned to groups with approximately balanced sample sizes. The animals were excluded upon they reach to the humanitarian endpoint.

### Hepatitis B vaccination

Each mouse underwent a single intramuscular vaccination, receiving either the Hepatitis B vaccine (2 µg HbsAg, Beijing Tiantan Biological Products Co., Ltd., Beijing, China) or an equivalent volume of sterile phosphate-buffered saline (0.1 ml), following the procedures detailed in our previously reported protocol. This specific dosage has been established for its efficacy in inducing adaptive immune responses and cytokine production in the periphery.^7^ Importantly, no discernible differences were observed in the overall behaviour, including grooming and autonomic activities, between the vaccinated and control mice.

### Adoptive transfer

After 7 days following the previous HBV injection, the spleen of donor mice was collected and homogenized into a single-cell suspension using RPMI Medium 1640 Basic (Life Technologies, Beijing, China) supplemented with 10% foetal bovine serum. ACK Lysis Buffer lysed red blood cells. The splenocytes were resuspended in 0.5 × 10^8^ cells/ml of PBS. Following the manufacturer's instructions, splenocytes were further isolated by negative selection for CD3^+^ T cell enrichment using the EasySep T cell isolation kit (Stem Cell Technologies, Vancouver, BC, Canada). Immunodeficient nude mice (Naive mice) were reconstituted with 10 million cells in a total volume of 200 µl PBS via tail vein injection. Recipient Naive nude mice received CD3^+^ T cells from either HBV-immunized (thereafter, HBV to Naive) or control mice (PBS to Naive) or Naive mice and were behaviourally tested starting 2 weeks after the injection.

### Behavioural tests

#### Open-field test (OFT)

The OFT was conducted in a 40 × 40 × 40 cm white plastic box with overhead white lighting (∼100 lux). At the start of the test, mice were put in the middle of the open field arena and given 5 or 10 min to wander around. The mice's movements were captured on camera by an above unit. Clever Sys, Inc.'s Topscan 3.0 was used to analyse the travel distance. The open-field equipment was sanitized by 70% ethanol to minimize olfactory cues.

#### Elevated plus maze (EPM)

On the same day, the OFT task was followed by the EPM task for the mice. The mice were each always given a single spot in the middle with an open arm, and they had 5 min to wander around the device at will. A video camera mounted above the animal captured its activity on camera. Topscan 3.0 was used to examine the travel distance and velocity, duration and number of entries in the open and closed arms. The equipment was sanitized just like in the OFT.

### Flow cytometry

#### Spleens

One day after behavioural testing, mice were perfused with pre-cold PBS before tissue extraction. Different sets of animals’ spleens were harvested and prepared as above. After blocking with anti-Fc CD16/32 (1:100; Clone: 2.4G2, 553141, BD Biosciences). Samples were labelled with PE-conjugated anti-CD3 (0.25 µg/million cells; Clone: 17A2, 12-0032-82, eBioscience), APC-conjugated anti-CD3(0.1 µg/million cells; Clone: 1F4, 557030, BD Biosciences), PE-Cy5.5-conjugated anti-CD4 (0.06 µg/million cells; Clone: RM4-5, 35-0042-82, eBioscience), FITC-conjugated anti-CD8a (0.2 µg/million cells; Clone: 53-6.7, 561966, BD Biosciences), Brilliant Violet 421-conjugated anti-CXCR6 (0.12 µg/million cells; Clone: SA051D1, 151109, Biolegend), APC-conjugated anti-CD45 (0.06 µg/million cells; Clone: 30-F11, 559864, BD Biosciences), PE-conjugated anti-IFN-γ (0.125 µg/million cells; Clone: XMG1.2, 12-7311-41, eBioscience); PE-Cy7-conjugated anti-TNF-α (0.125 µg/million cells; Clone: MP6-XT22, 25-7321-80, eBioscience), APC-conjugated anti-CD44 (0.14 µg/million cells; Clone: IM7, 559250, BD Biosciences) and APC-conjugated anti-CD62L (0.14 µg/million cells; Clone: MEL-14, 553152, BD Biosciences). CD44 and CD62L (L-selectin) surface markers were used to define three major subsets of CD8^+^ T cells in mice: naive (CD44^low^CD62L^high^), central memory (CD44^high^CD62L^high^) and effector/memory (CD44^high^CD62L^low^). For intracellular staining of IFN-γ, cells were incubated with phorbol 12-myristate 13-acetate (PMA; 10 ng/ml; Sigma-Aldrich) and ionomycin (250 ng/ml; Sigma-Aldrich) for 6 h, and brefeldin-A (10 μg/ml; Sigma-Aldrich) was added for the last 4 h of incubation according to the intracellular fixation and permeabilisation buffer set (88-8824-00, eBioscience^TM^). Flow cytometry was done on CytoFLEX S mice and PBS mice using a Becton Dickinson FACS Aria III. The isolated single cells were collected in PCR tubes with lysis buffer and promptly stored at −80°C for subsequent RNA-sequencing analysis. This methodology closely adhered to the established procedures outlined in previous research.^9^

#### Brains

According to our previously reported protocol,^10^ brains were quickly removed, and meninges (dura mater, arachnoid mater) were carefully stripped from the exterior surfaces after perfusion of pre-cold PBS. The brain was rapidly dissected and immersed in cold Hank’s balanced salt solution (HBSS). The brain was minced by scissors and homogenized using a Dounce homogenizer in ice-cold HBSS 30 times without bubbles. The cell suspension was then transferred to a pre-chilled 15-ml tube and filtered through a prewet (HBSS) 70-μm nylon mesh. Myelin and debris were removed using a modified cold 40% Percoll (Merck Millipore, 17-0891-02) gradient by centrifugation for 30 min at 500 g with no acceleration or braking.^10^ All samples were filtered through a 70-μm nylon mesh and blocked with anti-Fc CD16/CD32 (1:100; Clone: 2.4G2, 553141, BD Biosciences). The following fluorochrome-labelled monoclonal antibodies were used for experiments: PE-conjugated anti-CD3 (0.25 µg/million cells; Clone: 17A2, 12-0032-82, eBioscience); PE-Cy5.5-conjugated anti-CD4 (0.06 µg/million cells; Clone: RM4-5, 35-0042-82, eBioscience); FITC-conjugated anti-CD8a (0.2 µg/million cells; Clone: 53-6.7, 561966, BD Biosciences); PE-conjugated CD45 (0.06 µg/million cells; Clone: I3/2.3, 567111, BD Biosciences), FITC-conjugated CD11b (0.06 µg/million cells; Clone: M1/70, 561688, BD Biosciences), APC-conjugated NK-1.1 (0.06 µg/million cells; Clone: PK136, 561117, BD Biosciences). Samples were analysed by Beckman CytoFlex S and CytExpert software (Beckman).

#### Immunofluorescence

After harvesting the spleen, the brains of mice were excised and subsequently fixed overnight in 4% paraformaldehyde at 4°C and dehydrated with 10–30% sucrose at 4°C for 72 h. The brains were sliced into serial coronal sections (40 µm) on a freezing microtome (Leica SM2000 R; Leica Microsystems GmBH, Wetzlar, Germany). For 5-bromo-2′-deoxyuridine (BrdU) staining, mice were administered a single intraperitoneal (i.p.) injection of 50 mg/kg of BrdU once daily, and samples were harvested 2 days after the first injection. The following primary antibodies were used: rat anti-mouse anti-CD3 (1:400; Clone: 17A2, 14-0032-82, eBioscience, CA, USA), rabbit anti-CD4 (1:400; Clone: pSer^433^, SAB4504134, Sigma-Aldrich, St. Louis, MO, USA), rat anti-CD8a (1:400; Clone: 4SM15, 14-0808-82, eBioscience, CA, USA), rat anti-CD68 (1:400; Clone: FA-11, MCA1957, Bio-Rad), rat anti-BrdU (1:400; Clone: BU1/75 (ICR1), ab6326, Abcam), rat anti-Ki67 (1:400; Clone: SolA15, 14-5698-80, eBioscience, CA, USA), rabbit anti-DCX (1:500; ab18723, Abcam, Cambridge, UK), mouse anti-NeuN (1:400; Clone: 1B7, ab104224, Abcam, Cambridge, UK), rabbit anti-Iba-1 (1:2000; 019-19741, Wako Chemicals, Japan), mouse anti-GFAP (1:1000; Clone: CL2713, AMAB91033, Sigma-Aldrich, St. Louis, Missouri, USA), PE Rat anti-Mouse CXCL16 (1:400, Clone: 12-81, 566740, BD Biosciences). The following secondary antibodies were used: Alexa Fluor 488 goat anti-rabbit (1:1000, A-11034, Invitrogen), Alexa Fluor 488 donkey anti-rat (1:1000, A48269, Invitrogen), Alexa Fluor 555 goat anti-rat (1:1000, A-21434, Invitrogen), Alexa Fluor 488 goat anti-mouse (1:1000, A-11029, Invitrogen) and Alexa Fluor 594 donkey anti-rat antibodies (1:1000; A-21209, Invitrogen).

#### Smart-seq2 single cell RNA sequencing

We sorted 300 CD8^+^ T single cells from the abovementioned harvested spleens and kept in lysis buffer at −80°C for transcriptome analysis using Illumina NovaSeq 6000. (Shenzhen E-Gene Technology Co., Ltd.). Single cells should be harvested promptly, within 30 min, and immediately placed in a lysis buffer containing a ribonuclease inhibitor. This step is crucial as it effectively blocks RNA degradation and stabilizes the RNA for subsequent analysis.^9^

#### Quantitative real-time PCR (qPCR)

Total RNA from the mouse spleen was extracted using the RNAprep pure cell/bacteria kit (Tiangen Biochemical Technology, Beijing, China). Total RNA (total RNA) (2.5 μg) was converted to cDNA using the PrimeScriptTM RT Master Mix cDNA Reverse Transcription kit (Takara Biomedical Technology Co., Ltd., China). The expression of a specific mRNA was measured using a fluorescence-based quantitative real-time PCR (qPCR). Quantitative PCR reactions were performed for each sample using TB Green® Premix Ex TaqTM II (Takara Biomedical Technology Co., Ltd., China). β-Actin was chosen as the reference gene because of its stable expression in the spleen. Amplification cycles were 95°C 30 s and 40 cycles of 95°C 5 s and 60°C 30 s. At the end of the assay, melting curves in the 65–95°C range were constructed to assess the specificity of the reaction. All quantitative real-time PCR reactions were performed using the Bio-Rad CFX96 real-time PCR system and analysed using the comparative Ct method after normalisation to β-actin. All primer sequences are listed below: (Sequence from 5′ to 3′)

*Ptger4*-F: TTCTTCGGTCTGTCGGGTCTCAG

*Ptger4*-R: CTGTAGAAGTAGGCGTGGTTGATGG

*Arid5a*-F: ACGTGTATGATGAACTTGGCGGTAG

*Arid5a*-R: TGGTAGGAGGCAGTGGCTTGTC

*Mc1r*-F: CTCAACTCCAATGCCACCTCTCAC

*Mc1r*-R: CAGCACATTCTCCACCAGACTCAC

*CXCR6*-F: CTCTCTCACCTGGGAGTCTCAAGTC

*CXCR6*-R: GTCTCTACATTGTGGGAGGCAGAA

*β-actin-*F: GTGCTATGTTGCTCTAGACTTCG

*β-actin*-R: ATGCCACAGGATTCCATACC

### CD8 T cell ablation and injection of 5-bromodeoxyuridine (BrdU)

Depletion of CD8^+^ T cells in 4-6-week-old C57BL/6 mice was achieved by injecting In*Vivo*MAb anti-mouse CD8α (BE0061, Clone: 2.43, BioXcell) or rat IgG2b isotype antibody (BE0090, Clone: LTF-2, BioXcell) given i.p. at 100 mg/mouse for three consecutive days, (i.e. Days 0, 1, and 2), and hepatitis B vaccine was administered on Day 3 with a booster injection of anti-CD8α antibody on Day 4, followed by maintenance through weekly injections.^11-13^ This consisted of an injection of the hepatitis B vaccine on Day 3 and a booster injection of anti-CD8α antibody on Day 4. Two weeks following the intramuscular injection of the HBV, BrdU was administered once intraperitoneally at a dosage of 0.05 ml/dose (50 mg/kg). The mice were subsequently sacrificed, spleens harvested, and immune cells prepared to control for the absence of CD8^+^ T cells by using flow cytometry with APC-conjugated anti-CD3 (0.1 µg/million cells; Clone: 17A2, 14-0032-82, eBioscience™), PE-Cy5.5-conjugated anti-CD4 (0.06 µg/million cells; Clone: RM4-5, 35-0042-82, eBioscience), FITC-conjugated anti-CD8a (0.1 µg/million cells; Clone: 53-6.7, 561966, BD Biosciences) and APC-R700-conjugated anti-CD45 (0.06 µg/million cells; Clone: 30-F11, 565478, BD Biosciences).

### Brain stereotactic injection of the CXCL16 antibody

Neutralisation of the chemokine CXCL16 in the mouse brain by stereotactic injection of the CXCL16 antibody via the lateral ventricle (LV) intracranial injection, 4–6-week-old C57BL/6 mice were anaesthetized with tribromoethanol and terteropentanol (1.25% solution, 0.2 ml/10 g, intraperitoneally). Then, the mice were placed in a stereotactic device (RWD Life Technology Co., Ltd., Shenzhen, China), and their body temperature was kept constant. A scalp incision on the surface of the skull and fontanel were exposed. We injected 4 μl of PE rat anti-mouse CXCL16 antibody (Clone: 12–81, 566740, BD Biosciences) or isotype control antibody Rat IgG1, κ (553925, BD Bioscience) into the left LV (AP, −0.22 mm from bregma, ML, +1.0 mm, DV, −2.25 mm from the brain surface) with a micro syringe (rate of infusion, 0.4 µl/min), and slowly withdrew the needle after 5 min to prevent fluid from refluxing. The skin was sutured, and the mice were warmed (33–34°C) by an electric heating pad to allow for recovery and then returned to the cages. Each mouse was injected with 0.1 ml of Hepatitis B vaccine on the following day. The right LV was injected of anti-CXCL16 antibody again on Day 7, as described previously.

### Statistical analysis

All results are expressed as mean ± SEM. An unpaired two-tailed Student’s *t*-test (between two groups) or one-way or-two-way ANOVA (Bonferroni or LSD comparison tests) or non-parametric test. Data distribution was assumed to be normal; this was also tested with histograms. Analysis and graphing were performed by GraphPad Prism version 10.1.1. *P* < 0.05 was considered statistically significant.

## Results

### HBV induces the proliferation of specific T cell subsets in wild-type mice

We have previously reported that HBV promotes hippocampal microglia activation and elevates pro-inflammatory cytokines TNF-α and IL-6 levels in the brain.^14^ Therefore, we further evaluated those microglia expressed more lysosomal protein, reflected by CD68 immunoreactivity in the hippocampus, which were correlated with hippocampal inflammation in HBV-vaccinated mice as compared with their controls, PBS-injected mice (Supplementary Fig. 1A–D). One clinical trial documented that HBV was associated with an autoimmune disease in a small minority of subjects,^15^ and cytotoxic CD8^+^ T cells play a vital role in virus-induced neuroinflammation.^16,17^ Here, our data also showed that CD8^+^, not CD4^+^ T cells were detected in the third ventricle adjacent to the brain parenchyma in HBV mice, but not in PBS-treated mice (Supplementary Fig. 1E–L). We also found that the proportion of peripheral CD8^+^ T cells was increased in the mice after HBV (Supplementary Fig. 1M–O).

**Figure 1:**
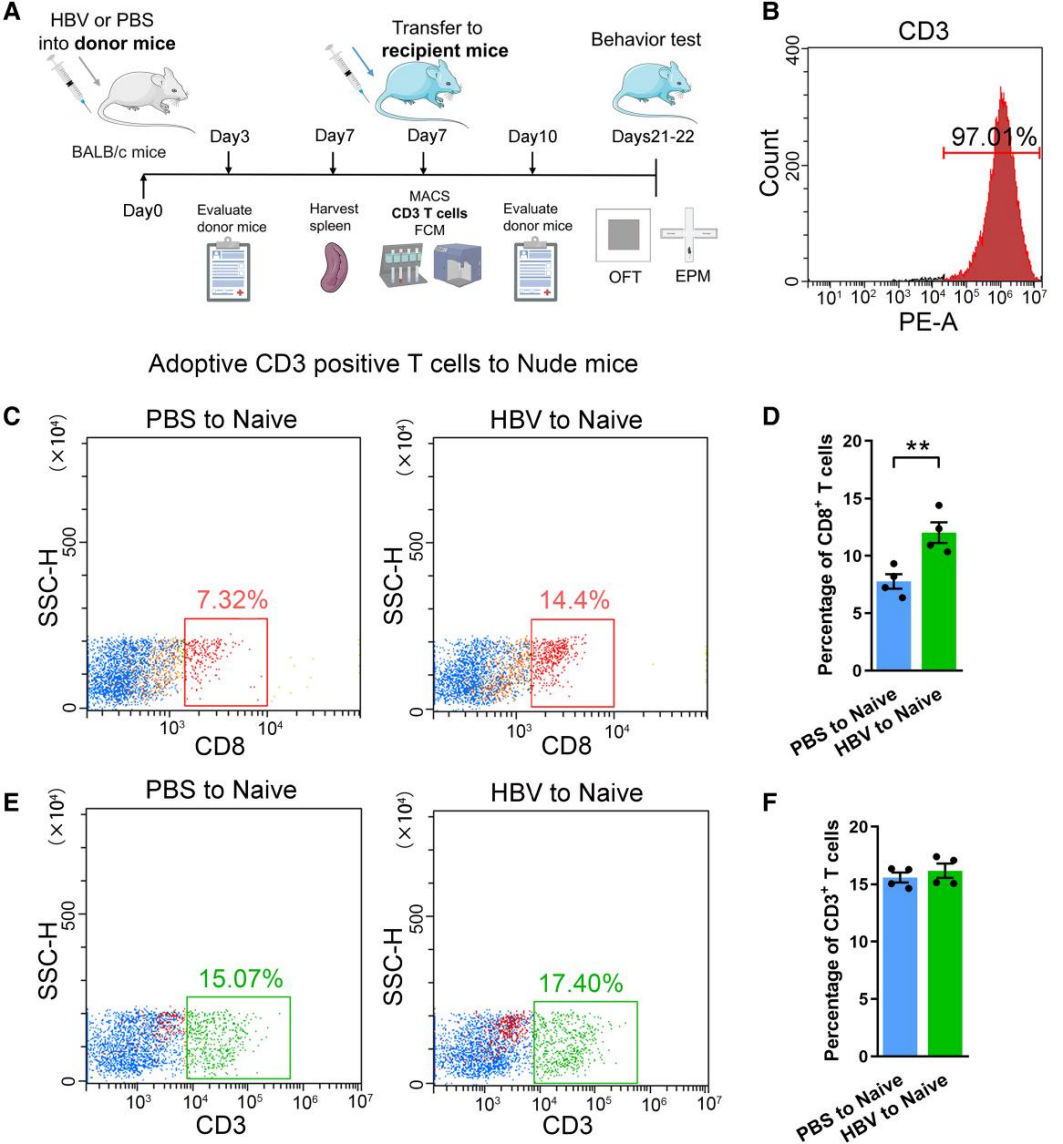
**CD8^+^ T cell recruitment in the immunodeficient mice after adoptive transfer CD3^+^ T cells.** (**A**) Schematics of the timeline of MACS-CD3^+^ T cell sorting from the spleen in HBV-induced mice and PBS-induced mice. CD3^+^ T cells were sorted from the spleen and transferred to nude mice on Day 7, and OFT and EPM tests were performed on Days 21 and 22 to assess the anxiety-like behaviour; MACS, magnetic-activated cell sorting; FCM, flow cytometry; OFT, open-field test; EPM, elevated plus maze. (**B**) The purified CD3^+^ T cells reached a high purity of 97.01%. (**C**) Representative images of adoptive transfer of HBV-induced CD3^+^ T cells (HBV-T cells) into immunodeficient nude mice. (**C** and **E**) Following the adoptive transfer of CD3^+^ T cells from HBV mice or PBS mice (HBV to Naive, PBS to Naive), the representative flow images of CD8^+^ T cells (**C**) and CD3^+^ T cells (**E**) of the total CD45^+^ immune cells. (**D** and **F**) Quantitative analysis of the percentage of CD8^+^ T cells and CD3^+^ T cells in the total immune cells. Student’s *t*-test (two-tailed), **P < 0.01, *n* = 5. Each dot on the dot plot represents one mouse.

To further validate the functions of HBV-induced T cells in this process, we adoptive HBV-induced CD3^+^ T cells to immunodeficient mice (as refereed HBV-Naive cells). A following flow cytometry test showed the purity of isolated CD3^+^ T cells were 97%, which indicated a high efficiency of the negative isolation kit (Fig. 1A and B). To gain further evidence about the lymphocyte infiltration and innate immune cell compartment in the central nervous system (CNS) after adoptive transferring CD3^+^ T cells, we performed a flow cytometry study on brain homogenate from HBV-Naive or PBS-Naive mice, to assess the proportion of CD4^+^ and CD8^+^ T cell subsets, CD11b^+^/CD45^low^ microglia, CD11b^+^/CD45^high^ macrophages, and NK cells. Our findings revealed that only the proportion of CD8^+^ T cells, rather than CD4^+^ T cells, and CD3^+^ T cells, was significantly increased in the brain of HBV-Naive mice relative to the controls (Fig. 1C–F; Supplementary Fig. 2C and D). About the innate immune cells, no significant changes were detected in monocyte-derived macrophages, microglia and NK cells between HBV-Naive mice and PBS-Naive mice (Supplementary Fig. 2A, B, E and G).

Next, the neuroanatomical localisation of T cells was analysed by immunostaining with the specific anti-CD3 and anti-CD8 antibodies. CD3^+^ T cells, including CD8^+^ T cells, were mainly observed in the third ventricles (Supplementary Fig. 3A–C), the choroid plexus of the LV (Supplementary Fig. 3D), brain parenchyma around the meninges (Supplementary Fig. 3E) and brain parenchyma (Supplementary Fig. 3F). As dural sinuses are recognized as a neuroimmune interface bridging the periphery and the brain, where patrolling T cells serve a vital role in immune surveillance.^18^ Expectedly, our data also showed that more CD8^+^ T cells were recruited to the dura mater of in HBV-Naive mice, relative to PBS-Naive mice (Supplementary Fig. 4A–C). Furthermore, we observed that CD8^+^ T cells can directly pass through blood vessels in the dura mater and transverse sinus (Supplementary Fig. 4D), indicating a potential pathway for migration of the CD8^+^ T cells to the brain. Taken together, these data indicated that HBV can promote peripheral CD8^+^ T cell migration into the brain.

### Peripheral CD8^+^ T cells affect anxiety behaviour and neurogenesis

We assumed that the altered peripheral CD8^+^ T cells may migrate to the CNS, regulating emotional status and neuronal cell proliferation. To further explore the role of CD8^+^ T cells in regulating brain functions, we analysed the changes in the emotional status of the mouse using the OFT and the EPM test and analysed the newborn neuronal counts by immunostaining BrdU and DCX.

**Figure 2:**
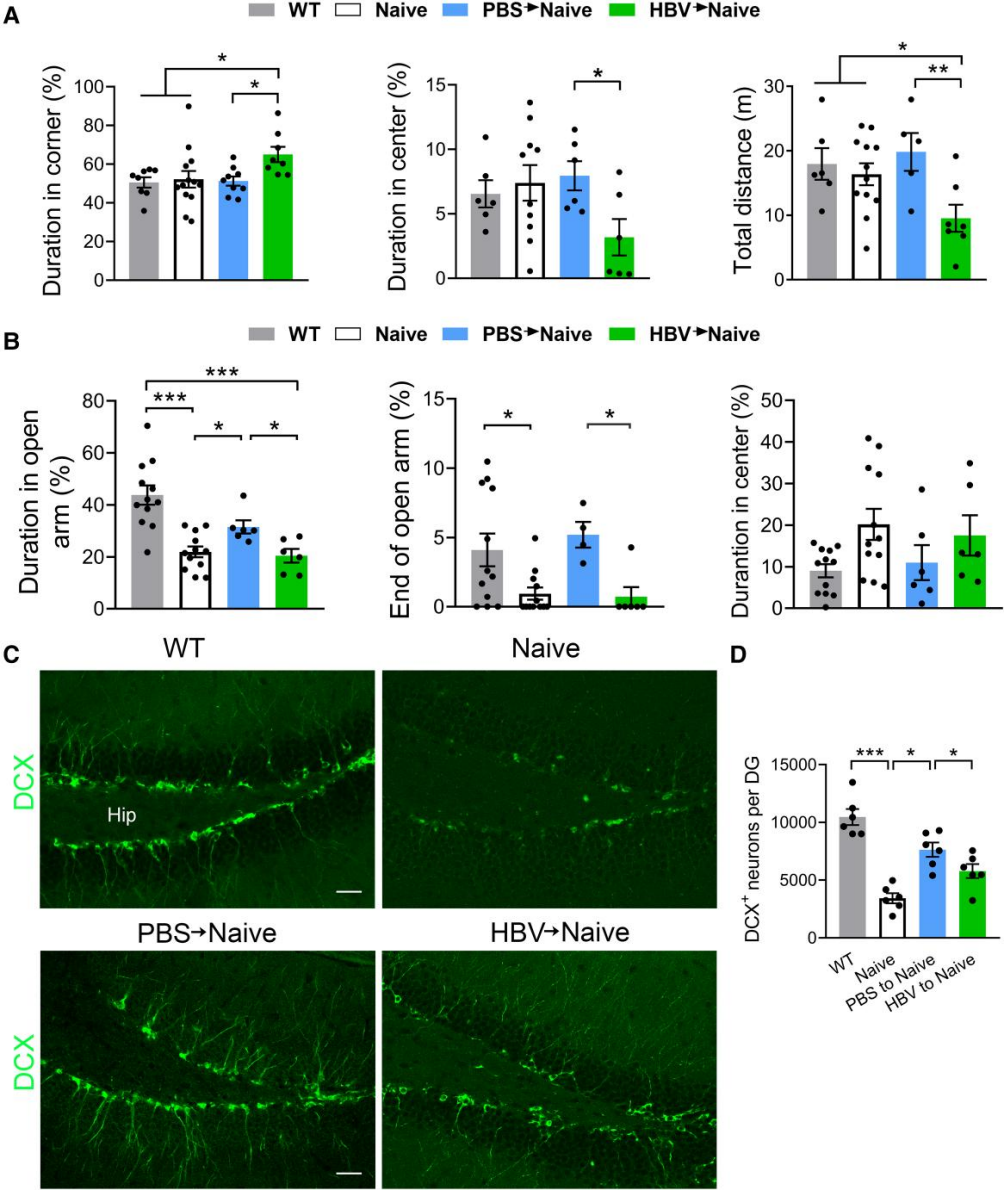
**Transfer of CD3^+^ T lymphocytes from HBV imitates anxiety-like behaviour in HBV-vaccinated mice.** (**A**) Following the adoptive transfer of CD3^+^ T cells from HBV mice to the immunodeficient nude mice (HBV-T cells to Naive) on a BALB/C background, had less spent time in the centre area and more time in the corner in the OFT, less time in the open arm and its end (head of the open arm) in the EPM test than in PBS-T cells to Naive mice (**B**). Transfer of CD3^+^ T cells from HBV mice decreases hippocampal DG cell proliferation. There were significantly fewer (25%) newborn cells in HBV to Naive mice than in PBS to Naive mice, which is comparable to that in WT mice. (**C**) Representative photomicrographs of the DCX-labelled cells in the DG of the groups are shown. (**D**) Quantitative analysis of DCX^+^ labelled neuroblast cell counts in the hippocampal DG region. Scale bar: 100 μm; one-way ANOVA, *P < 0.05, **P < 0.01, ***P < 0.001, *n* = 5. Each dot on the dot plot represents one mouse.

In the OFT, HBV-Naive mice travelled longer time in the corners, shorter time in the centre area, and had less total distance compared with those of PBS-Naive mice (Fig. 2A). No significant differences were found in these indexes from Naive mice, wild-type mice and PBS-Naïve mice in the OFT (Fig. 2A). Likewise, in the EPM test, HBV-Naive mice spent less time in the open arms and the end of the open arms than in PBS-Naive mice (Fig. 2B). Similar results were found in immunodeficient Naive mice (Fig. 2B). Furthermore, we examined whether transferred T cells affect neuronal cell proliferation in the hippocampus. We found that the fewest number of neuroblast cells labelled by DCX antibody were observed in the dentate gyrus of Naive mice compared with the other three groups (Fig. 2C and D). After adoptively transferring HBV-induced T cells, the DCX^+^ neuroblast cell count can be partially restored but was still significantly less than that of PBS-Naive mice, which reached to wild-type level (Fig. 2C and D). These results indicated that the adoptively transferring T cells from HBV mice may retain the immunological memory and imitate its anxiety-like and inhibitory neurogenetic effects.

### Transcriptional analysis of CD8^+^ T cells reveal specific genes participating in inflammatory cytokine secretion

**Figure 3:**
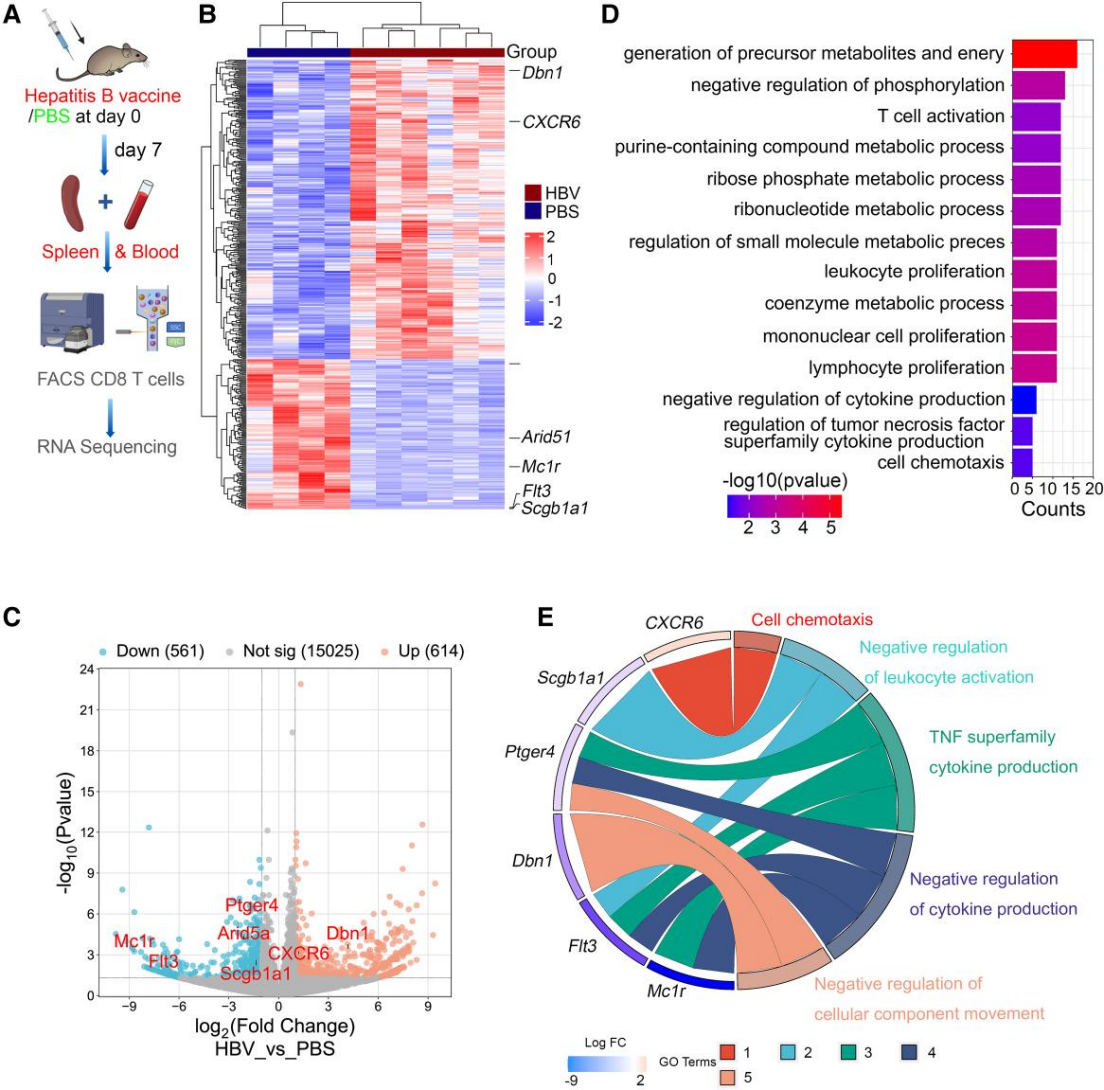
**HBV induces inflammatory cytokine response and in CD8^+^ T cells.** (**A**) Schematics of the experimental procedure, including vaccination, FACS CD8^+^ T cells, and RNA sequencing. (**B**) Heat map of differentially expressed genes from HBV-induced versus PBS-induced CD8^+^ T cells, *n* = 6 mice for the HBV group, *n* = 4 mice for the PBS group. (**C**) Volcano plots using common DEGs of HBV-induced and PBS-induced CD8^+^ T cells (*P* < 0.05, FDR < 0.05). (**D**) Top enriched biological process pathways from GO analysis based on these two groups: CD8^+^ T cells in HBV mice and PBS mice. (**E**) The GO chord showed a circularly composited overview of the changed genes, including *CXCR6*, *Arid5a*, *Scgb1a1*, *Ptger4*, *Mc1r* and *Flt3* and their assigned GO terms.

**Figure 4:**
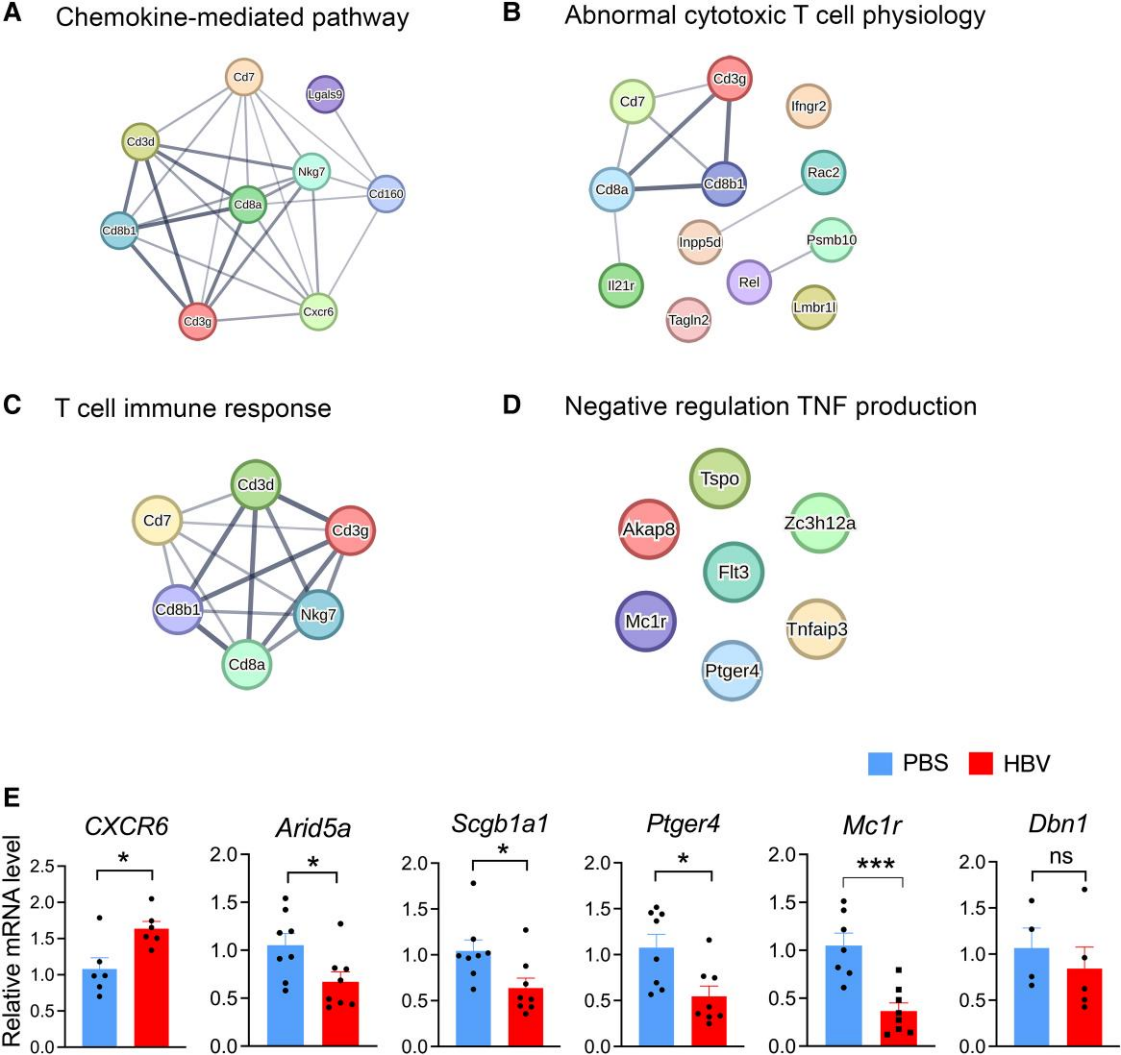
**HBV induces CD8^+^ T cell chemotaxis activation.** (**A–D**) String network analysis of the DEGs based on the enriched signal pathway showed chemokine-mediated response and T cell immune response (String enriched *P* < 0.05). (**E**) Quantitative PCR analysis was conducted to measure the expression of chemokine gene *CXCR6*, and cytokine production-related genes, including *Arid5a*, *Scgb1a1*, *Ptger4*, *Mc1r* and *Flt3* in isolated CD8^+^ T cells from the spleens of both HBV mice and PBS-treated mice, two-tailed Student’s *t-*test, *P < 0.05, **P < 0.01, ***P < 0.001, ns: non-significant. *n* = 6–8. Each dot on the dot plot represents one mouse.

Next, we hypothesized that peripheral CD8^+^ T cells with immunological memory can migrate into the CNS, leading to microglia activation by secreting inflammatory cytokines and thereby affecting brain function. To explore the differently expressed genes (DEGs) in CD8^+^ T cells, we extracted total RNA of isolated CD8^+^ T cells from HBV/PBS treated mice for RNA sequencing and analysed the candidate DEGs (Fig. 3A). We identified a significant increase of *CXCR6* gene expression in CD8^+^ T cells, as compared to the controls (Fig. 3B and C). Importantly, CD8^+^ T cells RNA-sequencing analysis also identified a significant reduction of cytokine regulatory genes in HBV mice compared to in PBS-treated mice, including *Arid5a*, *Mc1r*, *Flt3*, *Ptger4* and *Scgb1a1* (Fig. 3C). Gene ontology (GO) enrichment analysis showed that T cell activation, lymphocyte proliferation, negative regulation of cytokine production, regulation of TNF superfamily cytokine production and cell chemotaxis were enriched in HBV-induced CD8^+^ T cells (Fig. 3D). Moreover, GO Chord displayed that significantly changed genes in HBV-induced CD8^+^ T cells were assigned to cell chemotaxis, negative regulation of leukocyte activation, tumour necrosis factor superfamily cytokine production, negative regulation of cytokine production and negative regulation of cellular component movement (Fig. 3E). STRING interaction network analysis predicated chemokine-mediated pathway, abnormal cytotoxic T cell physiology, T cell immune response and negative regulation of TNF production based on the DEGs (Fig. 4A–D). In addition, these DEGs were further verified in the spleen tissue by RT-PCR test (Fig. 4E). Fluorescence-activated cell sorting (FACS) analysis of cells isolated from the spleen showed increased CXCR6^+^CD8^+^ T cells in HBV mice compared to that in PBS mice (Supplementary Fig. 5A–C). Together, these data show that HBV induced inflammatory signature in CD8^+^ T cells and enriched chemokine pathway and TNF family signalling.

### CD8^+^ T cells are required for HBV-induced decreased neogenesis and anxiety-like behaviour

**Figure 5:**
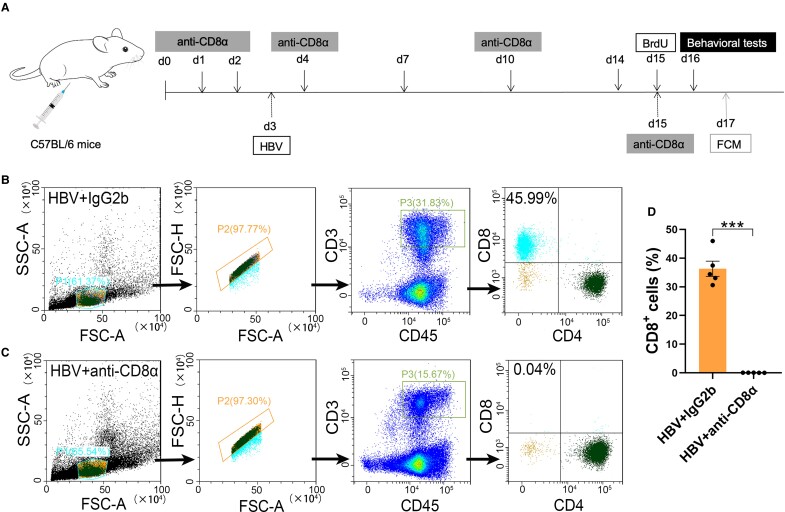
**Restoration of reduced newborn neurons in HBV-immunized mice after CD8^+^ T cell ablation.** (**A**) Deletion of CD8^+^ T cells pattern diagram by i.p. injection of anti-CD8α antibody. Anti-CD8α antibody was administered at 100 μg/mouse for 3 consecutive days starting on Day 0 (i.e. Days 0, 1, and 2), and hepatitis B vaccine was administered on Day 3 with a booster injection of anti-CD8α antibody on Day 4, followed by maintenance through weekly injections. Controls were injected with an isotype control antibody, IgG2b. BrdU was administered via a single i.p. injection at a dose of 0.05 ml (50 mg/kg). (**B** and **C**) Representative flow cytometry of splenic CD8^+^ T cells from mice immunized with HBV treated with anti-CD8α neutralizing antibody or IgG2b. (**D**) Quantitative analysis of CD8^+^ T cells showed a depletion efficiency of up to 99.9%. Student's *t*-test (two-tailed), *n* = 5 mice per group; ***P < 0.001. Each dot on the dot plot represents one mouse.

**Figure 6:**
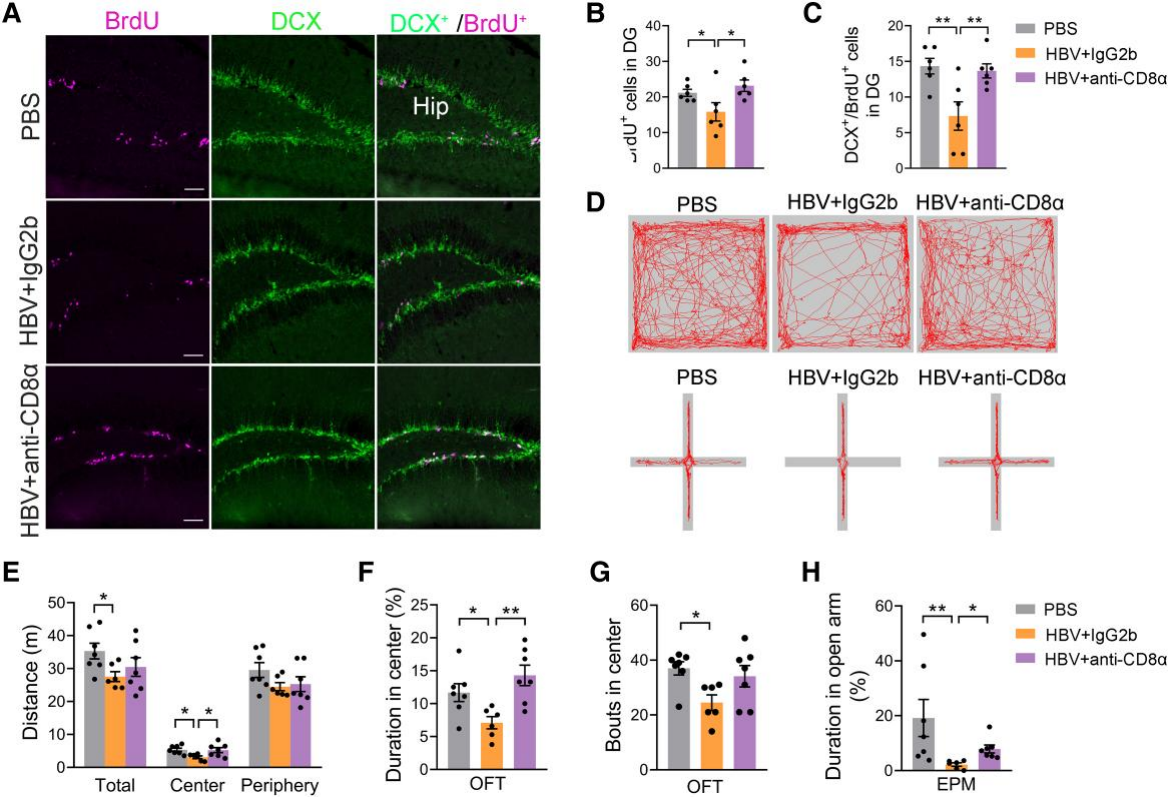
**Improvement of anxiety-like behaviour in HBV-immunized mice after CD8^+^ T cell ablation.** (**A**) Representative micrographs of BrdU and DCX of HBV-immunized mice treated with anti-CD8α neutralizing antibody or IgG2b isotype control antibody, and PBS-treated wild-type mice; Hip: hippocampus. (**B** and **C**) Quantitative analysis of BrdU^+^ (**B**) and BrdU^+^/DCX^+^ (**C**) labelled cell counts in the hippocampal DG region of the PBS, HBV + IgG2b and HBV + anti-CD8α neutralizing antibody groups. DG: dentate gyrus. (**D**) Representative maps of the OFT and the EPM. (**E–G**) In the OFT, anti-CD8α neutralizing antibody treatment increased the travel distance, and the time spent in the central area in HBV mice compared with the control mice. (**H**) In the EPM test, anti-CD8α neutralizing antibody treatment caused HBV mice to stay longer in the open arm compared with the control group. Scale bar: 50 μm in (**A**). (**B**, **C**, **E** and **F**) one-way ANOVA; (**G** and **H**) non-parametric test; *n* = 6–7 mice/group, *P < 0.05, **P < 0.01. Each dot on the dot plot represents one mouse.

To further demonstrate the function of CD8^+^ T cells induced by HBV to regulate brain function, i.e. to reduce neurogenesis and anxiety-like behaviour, we tested the neuronal proliferation in the hippocampus and anxiety-like behaviour in HBV mice after anti-CD8α neutralizing antibodies treatment. First, we verified that i.p. injection of anti-CD8α antibodies resulted in the 99% CD8^+^ T cell ablation in the spleen (Fig. 5A–D). Next, we observed CD8^+^ T cell ablation rescued the number of newborn cells (BrdU^+^) and newborn neurons (BrdU^+^/DCX^+^) in the hippocampus in HBV mice, reaching to the levels of PBS mice (Fig. 6A–C). For the behaviour test, CD8^+^ T cell ablation prevented HBV-mediated decrease in total distance, duration in the centre, and bouts in the centre of the OFT compared to IgG2b controls (Fig. 6E–G). Moreover, the percentage of time spent in the open arm in the EPM test was increased after anti-CD8α antibody treatment (Fig. 6D and H). Thus, these findings indicate that CD8^+^ T cells are required for decreased hippocampal neurogenesis and improvement of anxiety-like behaviour in HBV mice.

### Glial-derived CXCL16 recruits CD8^+^ T cells into the brain via CXCR6

**Figure 7:**
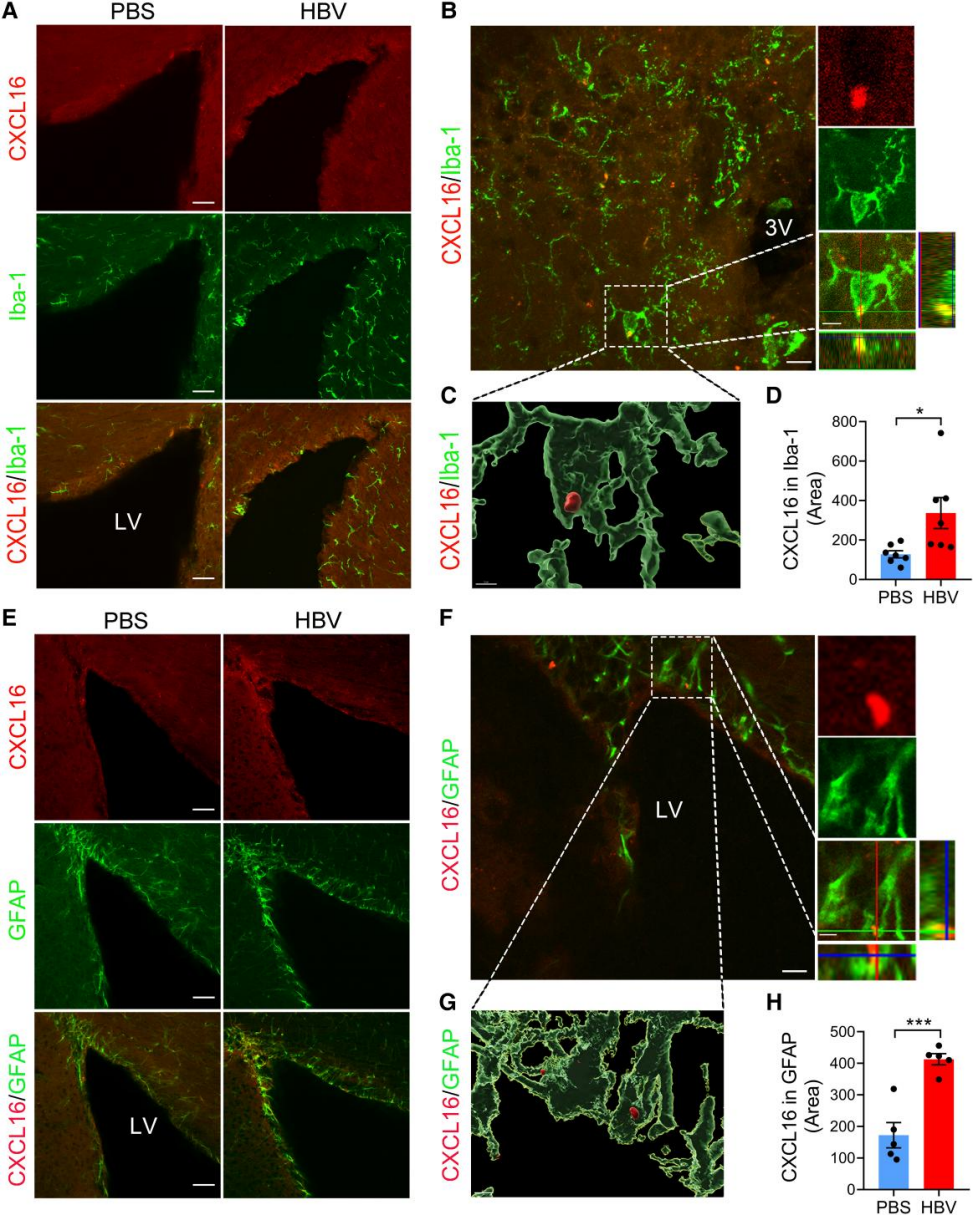
**Hepatitis B vaccine induces brain cells to secrete chemokine CXCL16.** (**A**, **B**, **E** and **F**) Representative photomicrographs of wild-type mice treated with HBV or PBS in the LV, and third ventricle stained for Iba-1, GFAP and CXCL16. Orthogonal image of CXCL16 expression in microglia and astrocyte in (**B**) (right) and (**F**) (right). (**C**) 3D reconstruction of CXCL16 signals in microglia from dotted line box in (**B**). (**G**) 3D reconstruction of CXCL16 signal in astrocyte from dotted line box in (**F**). (**D** and **H**) Quantitative immunofluorescence analysis of CXCL16/Iba-1, CXCL16/GFAP double labelling in both groups. Scale bar: 50 μm in (**A** and **E**), 10 μm in (**B** and **F**), 2 μm in (**C** and **G**). (**D** and **H**) Student's *t*-test (two-tailed), *n* = 5–7 brain slices from four mice, *P < 0.05, **P < 0.01, ***P < 0.001. Each dot on the dot plot represents one brain slice.

The convergence factor CXCL16 and its distinctive receptor CXCR6 are cell factors that regulate cellular directional migration in multiple disease status.^19,20^ Using immunofluorescence staining analysis, we found that 48.34% microglia, 44.66% astrocytes in the brain expressed CXCR6 ligand CXCL16 (Iba-1/CXCL16, GFAP/CXCL16, depicted in Fig. 7A–H). Furthermore, neurons have the restricted ability to express 7.00% of CXCL16 (Supplementary Fig. 6A–D; Supplementary Table 1). HBV significantly induced expression of CXCL16 both in the microglia and astrocytes in the hippocampus, relative to the PBS-treated controls (Fig. 7D and H). Together with the RNA-sequencing data and flow cytometry data revealed that CD8+ T cells expressed significantly higher levels of CXCR6, which predicted a heightened response to CXCL16 signalling in the brain ‘borders’, including choroid plexus, dura mater and even the brain parenchyma. Therefore, the CXCL16-CXCR6 signalling pathway may be a potential molecular mechanism for CD8^+^ T cell recruitment to the brain in the context of HBV.

Blocking CXCL16/CXCR6 axis alleviates HBV-mediated decreased hippocampal neurogenesis and anxiety-like behaviour.

**Figure 8:**
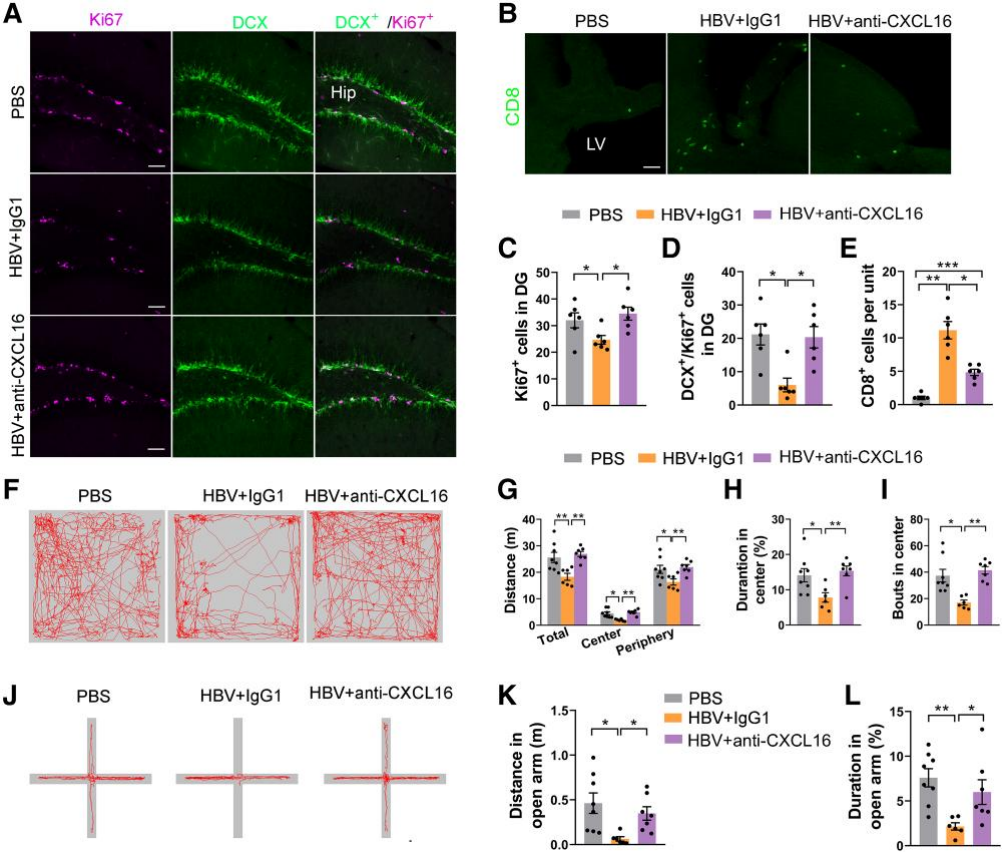
**The decrease in hippocampal neuronal proliferation and anxiety-like behaviour was improved after the CXCL16 blockade in HBV mice.** (**A**) Representative micrographs of Ki67 staining and DCX staining of HBV-immunized mice treated with anti-CXCL16 antibody or IgG1 isotype control antibody, and PBS-treated wild-type mice. (**B**) CD8^+^ T cells were detected in mouse ventricles after injection of anti-CXCL16 in the LV stained with CD8 and Hoechst. Reduction of CD8^+^ T cells in HBV mice after blockade of the chemokine CXCL16 in brain entry. (**C**) Quantitative analysis of Ki67 and DCX^+^/Ki67^+^ (**D**) labelled cell counts in the hippocampal DG region of the PBS, HBV + IgG1 and HBV + CXCL16 antibody groups. (**E**) Quantitative immunofluorescence analysis of CD8-labelled cells in brain ventricles of each group. (**F**) Representative maps of the OFT. (**G–I**) In OFT, anti-CXCL16 antibody treatment increased the activity distance, the percentage of stay time in the central region, and the percentage of entries into the central region in HBV mice compared with the HBV + IgG mice. (**J**) Representative maps of the EPM test. (**K**, **L**) In EPM, anti-CXCL16 antibody treatment resulted in HBV mice staying in open arms for longer periods and traveling distances than the control group. DG, dentate gyrus. (**C–E**, **G**, **H** and **K**) one-way ANOVA; (**I** and **L**) non-parametric test. Scale bar: 50 μm in (**A** and **B**); *n* = 6–8/group, *P < 0.05, **P < 0.01, ***P < 0.001. Each dot on the dot plot represents one mouse.

Next, we asked that how the CXCL16-CXCR6 axis mediates neurogenesis and its significance in regulating anxiety-like behaviours. After 2 weeks of injecting anti-CXCL16 neutralizing antibodies, we evaluated anxiety-related behaviours using the OFT and EPM tests to corroborate the possible roles of the CXCL16/CXCR6 axis in the recruitment of CD8^+^ T cells into the brain. The data showed that blocking CXCL16 signalling in the LV decreased CD8^+^ T cell recruitment to the brain compared to the IgG1 control mice (Fig. 8B and E). Importantly, significantly more Ki67^+^/DCX^+^ newborn neurons in the hippocampus were observed (Fig. 8A, C, and D). After treatment with CXCL16 antibodies, HBV was not able to decrease the distance travelled, duration, or number of entries into the central area (Fig. 8F–I) in OFT, or the duration and distance in the open arm in EPM (Fig. 8J–L). These findings indicate that the CXCL16/CXCR6 axis plays crucial roles in HBV-mediated CD8^+^ T cell migration into the brain, thereby inhibiting hippocampal neurogenesis and leading to anxiety-like behaviours.

### HBV increases CD8 T effector cells and increases TNF-α levels

**Figure 9:**
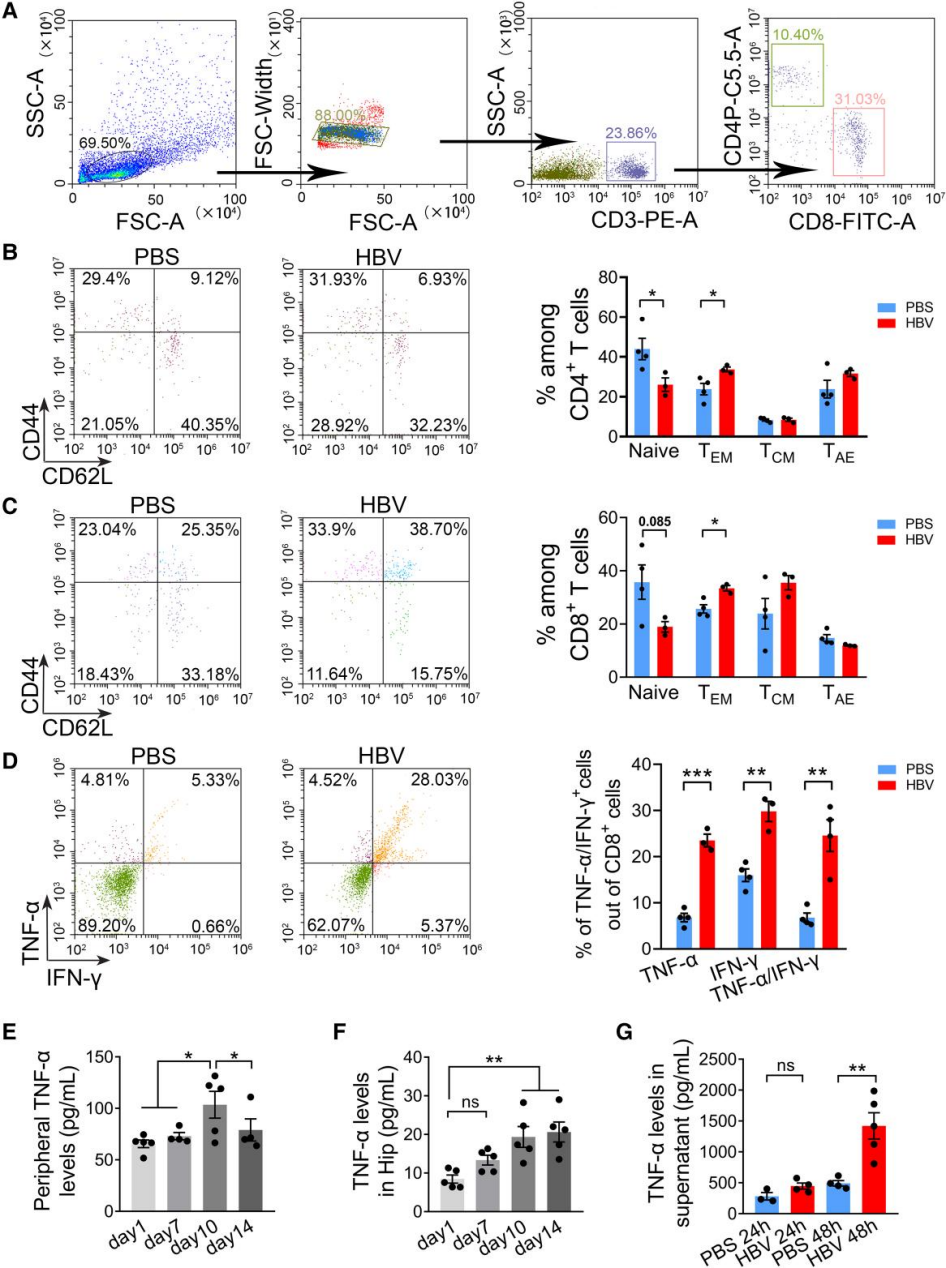
**HBV affects T cell subpopulation change and TNF-α secretion.** (**A–C**) Different activation status of CD4^+^ (**B**) and CD8^+^ (**C**) T cells in the spleen of WT mice after HBV vaccination. (**D**) Quantitative analysis of the percentage of CD8^+^ T cells-deriving TNF-α and IFN-γ in the spleen between HBV-vaccinated mice and the control group. (**E**) Serum TNF-α levels at Days 1, 7, 10 and 14 after HBV treatment. (**F**) Hippocampal TNF-α levels at Days 1, 7, 10 and 14 after HBV treatment. (**G**) TNF-α levels in the supernatant of primary spleen lymphocytes after HBV stimulation. (**B–D**) Student’s *t*-test (two-tailed), *n* = 3–4 mice/group, (**E**, **F**) one-way ANOVA, *n* = 3–5 mice/group, *P < 0.05, **P < 0.01. Each dot on the dot plot represents one mouse.

CD8^+^ T-cells can differentiate into distinct subpopulations based on the functions, and the expression of cell surface receptor molecular CD44 and CD62L.^21,22^ Recent studies have shown that the recruitment of clonal CD8^+^ T effector memory cells into the brain is linked to cognitive impairment, including aging and AD.^23,24^ We further explored the subset distribution of peripheral T cells and cytokine levels after HBV treatment. Our flow cytometry data showed that effector memory (CD62^low^ and CD44^high^) CD4^+^ T cell counts (Fig. 9A and B) and CD8^+^ T cell counts (Fig. 9A and C) were significantly higher in HBV mice compared to in PBS mic. Meanwhile, the counts of central memory (CD62^high^ and CD44^high^) and acute/activated effector (CD62^low^ and CD44^low^) CD4^+^ T cells (Fig. 9B) and CD8^+^ T cells (Fig. 9C) were comparable between the two groups. These results showed that peripheral effector memory CD4^+^ and CD8^+^ T cells may participate in this regulation (Fig. 9B and C). Given our earlier findings, which demonstrate that CD8^+^, but not CD4^+^ T cells have the ability to infiltrate the brain and regulate neuronal proliferation, we further evaluated TNF-α and/or IFN-γ^+^ cells among CD8^+^ T cells, and found that the percentage of TNF-α^+^, IFN-γ^+^ and TNF-α^+^/IFN-γ^+^ CD8^+^ T cells of total CD8^+^ T cells were significantly higher in HBV mice than that in PBS mice (Fig. 9D). These results were further supported by ELISA assay for a serologic test (Fig. 9E) and supernatant test to TNF-α (Fig. 9F and G). Notably, we found a significant increase in TNF-α levels in the hippocampus after HBV treatment. Together, these results suggest that CD8^+^ T cells are required for the increase in brain TNF-α levels after immunisation.

### TNF-α mediates HBV-induced decreased neurogenesis and anxiety-like behaviour

**Figure 10:**
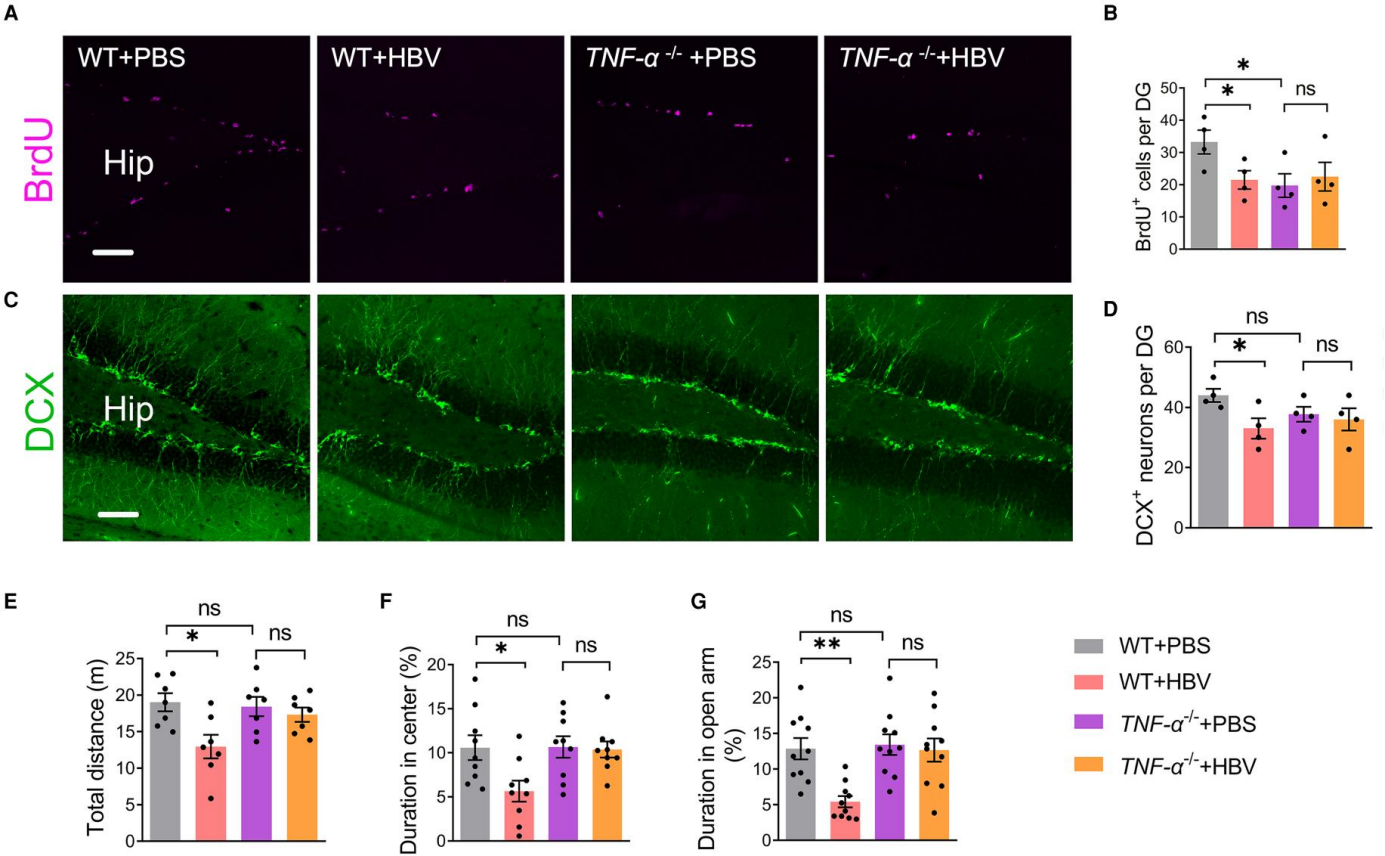
**TNF-α mediates decreased hippocampal neurogenesis and anxiety-like behaviour after HBV.** (**A** and **C**) Representative confocal images of newborn cells and neuroblast cells in the hippocampus of WT mice and *TNF-α* deficient mice that were injected with PBS and HBV, *n* = 4 mice per group; DG, dentate gyrus; (**B** and **D**) Quantification of newborn cells (BrdU^+^) and neuroblasts (DCX^+^) in the hippocampus from WT + PBS, WT + HBV, *TNF-α*^−/−^ +PBS, and *TNF-α*^−/−^ +HBV mice. (**E** and **F**) Total distance and duration in the centre area in OFT, *n* = 7–9 mice per group; (**G**) Duration in the open arm in the EPM test, *n* = 10 mice per group. (**B**, **D** and **E–G**) Two-way ANOVA; Scale bar: 50 μm in (**A** and **C**); *P < 0.05, **P < 0.01; ns, non-significant. Each dot on the dot plot represents one mouse.

We have shown that intracerebroventricular pumping of recombinant IFN-γ cytokines promotes hippocampal neuronal proliferation.^25^ Previous study has shown that intracellular TNF-α levels in the naive CD8^+^ T-cell cluster are obviously increased and negatively correlated with Montreal cognitive assessment scores in neurodegenerative disease.^26^ Interestingly, TNF-α pathway can potentially induce microglial activation in the prefrontal cortex in an acute paradoxical sleep deprivation-induced anxiety-like behaviours in mice.^27^ We assume that brain infiltrated CD8^+^ T cells might potentially inhibit hippocampal neurogenesis in HBV vaccinated mice. Therefore, our subsequent studies focused on the effects of the TNF-α derived from CD8^+^ T cells on neuronal proliferation and emotional state in mice. Using TNF-α deficient mouse model (which do not produce TNF-α), we further validate that HBV vaccination had no inhibitory effects on the hippocampal neurogenesis (Fig. 10A–D) and anxiety-like behaviour (Fig. 10E–G). Additionally, we observed that there are no significant differences in immature neurons, reflected by DCX^+^ cell counts in DG area between WT mice and *TNF-α* deficient mice (Fig. 10D). Taken together, these results suggest that the deleterious effects of HBV on hippocampal neurogenesis and anxiety-like behaviour are mediated by increased TNF-α production.

## Discussion

Vaccine hesitancy is a pressing public health concern that exposes individuals to preventable illnesses, posing a significant risk to public well-being. In our previous work with mice, we reported a transient decrease in hippocampal neurogenesis and the induction of anxiety-like behaviour following neonatal HBV.^7^ However, the underlying mechanisms remain elusive. Here, we explored that cellular and molecular mechanisms governing CD8^+^ T cells and TNF-α in the regulation of neurogenesis and behaviour after HBV administration. Our mechanistic investigations revealed that HBV induces the expression of glial-derived CXCL16, facilitating the trafficking of CD8^+^ T cells into the brain through CXCL16/CXCR6 signalling. Significantly, blocking CXCL16 in the brain or ablating peripheral CD8^+^ T cells mitigate the HBV-mediated downregulation of hippocampal neurogenesis and alleviates anxiety-like behaviour. Furthermore, using TNF-α deficient mice, we demonstrated that the diminished neurogenesis and anxiety-like behaviour observed after HBV vaccination require TNF-α derived from CD8^+^ T cells. These findings provide crucial insights into the intricate interplay between immune activation and neurobehavioural outcomes following HBV vaccination.

### HBV decreased hippocampal neurogenesis and anxiety-like behaviour via brain CD8 T cell accumulation

Previous studies have shown that CD8^+^ T cells migrate to the brain, and specific CD8^+^ T cell subsets have been linked to various conditions such as ageing,^28^ memory deficits,^29-31^ AD^24^ and neuropsychiatric complications.^32^ In alignment with these findings, our study revealed that both HBV and the adoptive transfer of HBV-induced CD3^+^ T cells prompted the migration of CD8^+^ T cell subsets into different brain regions, thereby reducing hippocampal neurogenesis and inducing anxiety-like behaviour. Furthermore, we observed a notable increase in effector memory CD8^+^ T cells in the spleen following HBV treatment. This observation aligns with existing studies indicating that elevated numbers of CD8^+^ T effector memory cells in older adults and AD patients may contribute to memory dysfunction and age-related chronic inflammatory diseases.^11,33,34^ Interestingly, several studies in individuals with chronic hepatitis B have reported that hepatitis B surface antigen (HBsAg)-specific CD8^+^ T cells play a role in antiviral effects through viral clearance.^35-37^ However, our data revealed that HBV, in contrast, reduced hippocampal neurogenesis and contributed to anxiety-like behaviour in mice, potentially attributed to the infiltration of HBV-induced CD8^+^ T cells in the brain.

### Glial-derived CXCL16 recruit and interact with CD8^+^ T cells into the brain via CXCR6

CXCL16, a chemokine belonging to the C-X-C family, plays a multifaceted role as a T-cell chemokine with diverse properties. Single-cell transcriptional profiling in cerebrospinal fluid (CSF) has revealed that the CXCL16-CXCR6 signalling pathway holds the potential to guide the entry of CD8^+^ effector memory T cells (T_EM_) into the brain CSF during healthy aging and cognitive impairment.^2^ Moreover, brain-resident memory CD8^+^ T cells (T_RM_), expressing CXCR6 and PD-1 and located in proximity to plaque-associated microglia, have been identified in human and mouse AD brains.^3^ Despite functional heterogeneity among different CD8^+^ T cell subsets in brain function, the chemotactic mechanism involving CXCL16 expressed by brain myeloid cells recruiting CD8^+^ T cells via CXCR6 remains conserved across models. Here, we also identified that the glial-derived chemokine CXCL16 interact with CXCR6, serving as a mechanism for CD8^+^ T cell entry into the brain following HBV treatment. Notably, our data revealed that microglia and astrocytes are the primary expressors of CXCL16 after HBV treatment, in contrast to the expression of CXCL16 by brain microglia and monocytes in neurodegenerative diseases. This finding is particularly significant in light of recent evidence suggesting that CXCL16/CXCR6 signalling plays a crucial role in brain-resident T cells, contributing to synapse elimination,^5^ and limiting further Aβ pathology and cognitive decline.^36^

### CD8^+^ T-cell infiltration inhibits neurogenesis and induces anxiety-like behaviour via TNF-α release

Under normal circumstances, immune cells located at the borders of the brain can remotely influence the function of CNS by releasing mediators such as cytokines and neurotrophic factors.^38-40^ CD8^+^ T cells, also known as cytotoxic T lymphocytes, play a pivotal role in anti-tumour immunity, antiviral immunity and neuronal dendritic spine plasticity, releasing various immune factors, including TNF-α and Granzymes.^41-44^ TNF-α, known for its significant pathological impact on the CNS, can affect various neurobehavioural processes,^45,46^ being produced by lymphocytes, endothelial cells, monocytes and neurons in the brain. Our findings consistently reveal that TNF-α, secreted by CD8^+^ T cells, inhibits neurogenesis and induces anxiety-like behaviour. This was further validated using TNF-α deficient mice. Additionally, CD8^+^ T cells secrete IFN-γ, another cytokine, after HBV, albeit in relatively small amounts. Flow cytometry data indicates that splenic CD8^+^ T cells secrete TNF-α at a 2.5-fold higher ratio than IFN-γ (Fig. 9D). Under physiological conditions, we have previously reported that IFN-γ enhances hippocampal neurogenesis^47^ and induces neuronal differentiation.^48^ Notably, a recent study unveiled that CD8^+^ T cell-induced IFN-responsive oligodendrocytes and microglia play a crucial role in modifying white matter aging.^49^ Thus, the impact of CD8^+^ T cell-produced IFN-γ on neurogenesis and anxiety-like behaviour in HBV mice remains to be understood. Additionally, whether and how brain CD8^+^ T cell accumulation interacts with microglial homeostasis,^50^ potentially impairing hippocampal neurogenesis, or directly regulating neuronal functions,^51^ remains elusive.

Collectively, our study highlights that the brain glial-derived CXCL16, promoting CXCR6^+^CD8^+^ T accumulation, acts on hippocampal neurogenesis and anxiety via TNF-α production. This neuroimmune axis provides an important mechanism through which peripheral immune activation, such as through vaccination, can modulate brain functions and anxiety.

### Limitation in this study

It is important to note that we lack direct evidence demonstrating that TNF-α derived from CD8^+^ T cells directly regulate neuronal proliferation in the hippocampus. We observed the infiltration of CD8^+^ T cells in various brain regions, including the choroid plexus adjacent to the lateral and third ventricles, the dura mater, and the brain parenchyma surrounding the meninges-regions identified as brain-border associated areas.^52^ Notably, the dural meninges exhibited a significant enrichment in immune cells and cytokines, constituting over 95% of the potential cytokine source in the meningeal space and the CSF.^24,39^ Consequently, cytokines produced by CD8^+^ T cells, such as TNF-α, have the potential to be released into the CSF. The dorsal hippocampal neurogenesis that we investigated is proximal to the third ventricle (as illustrated in https://atlas.brain-map.org/atlas). Therefore, it is conceivable that infiltrating CD8^+^ T cells may impede hippocampal neuron proliferation and modulate anxiety through the remote action of cytokine TNF-α.

## Supplementary Material

fcae315_Supplementary_Data

## Data Availability

The analysis for RNA-sequencing data were done by http://www.bioinformatics.com.cn/SRplot, an online platform for data analysis and visualisation.^53^ The DEGs for Heatmap and Volcano plots were uploaded as supplementary tables. The graphical abstract and the schematics of the timeline in Fig. 1A were created with BioRender.com (https://www.biorender.com/).
