# Supplementary material for: CD8 positive T-cells decrease neurogenesis and induce anxiety-like behaviour following hepatitis B vaccination
Source: Brain Commun. 2024 Sep 16;6(5):fcae315. doi: 10.1093/braincomms/fcae315 (PMC11462449; doi:10.1093/braincomms/fcae315)
Supplement: fcae315_Supplementary_Data [file fcae315_supplementary_data.docx]

Supplementary Figures and Tables


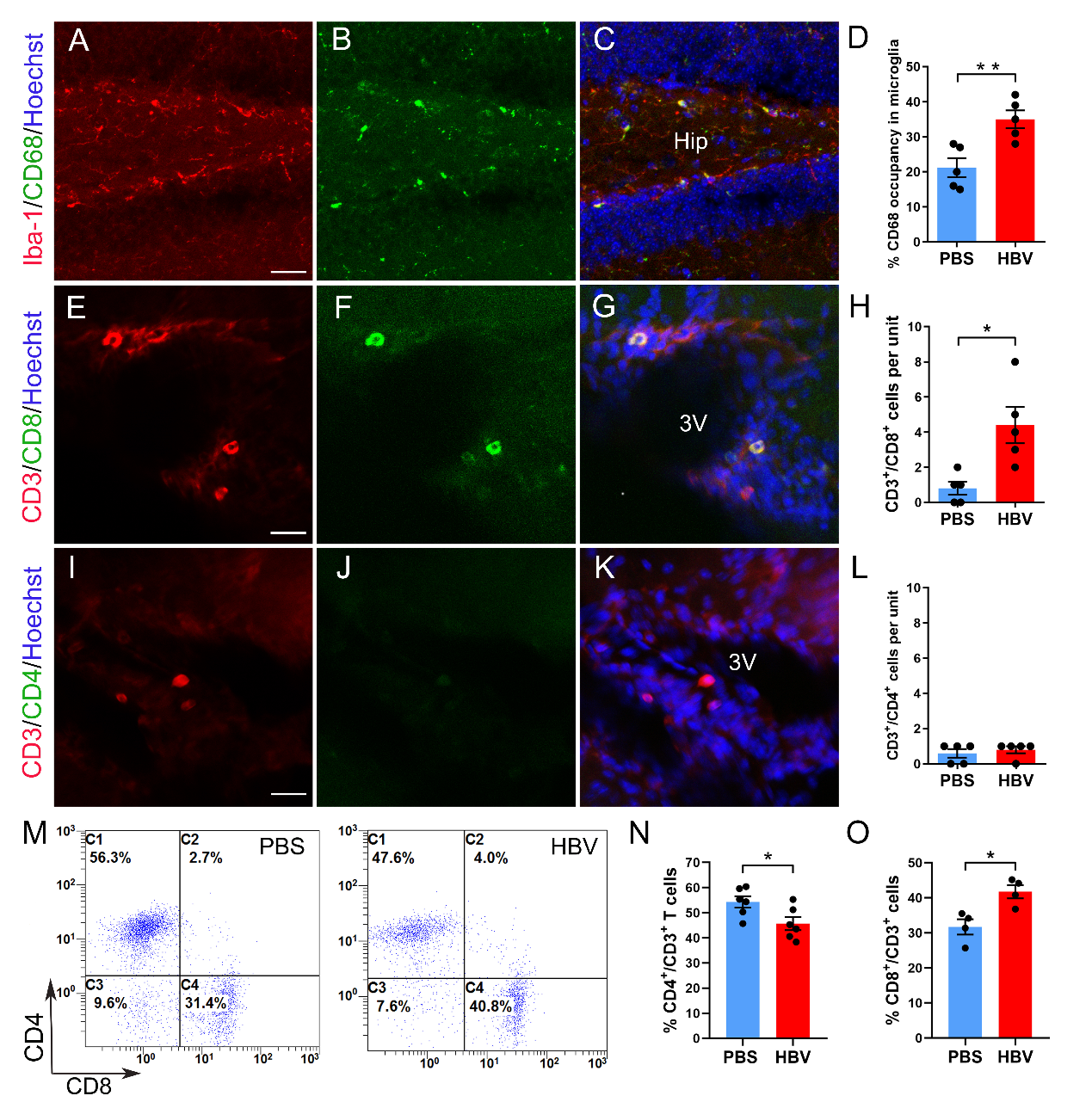


Supplementary Figure 1. HBV immunization induces microglia activation and T cell recruitment to the brain.

(A-C) Representative confocal images of the DG stained for Iba-1 (red) and with CD68 staining (green) from the wide-type mice treated with HBV or PBS. (E-K) Representative micrographs of the 3rd ventricle for CD3 (red) and CD8 staining (green, E-G), and CD3/CD4 (I-K). Quantification of Iba-1/CD68 (D), CD3/CD8 (H), and CD3/CD4 (L) double-labeled cell counts in the two groups. (M-O) Quantitative analyses of the number of CD4^+^ T cells (N) and CD8^+^ T cells (O) in the spleen of the PBS- group and the HBV group. Hip: hippocampus; 3V: third ventricle; DG: dentate gyrus. Scale bar: 100 μm in A-C; 50 μm in E-K. *n* = 5 brain slices from 3 mice per group; Student’s *t*-test (two-tailed), **P*<0.05; ***P*<0.01. Each dot on the dot plot represents one brain slice.


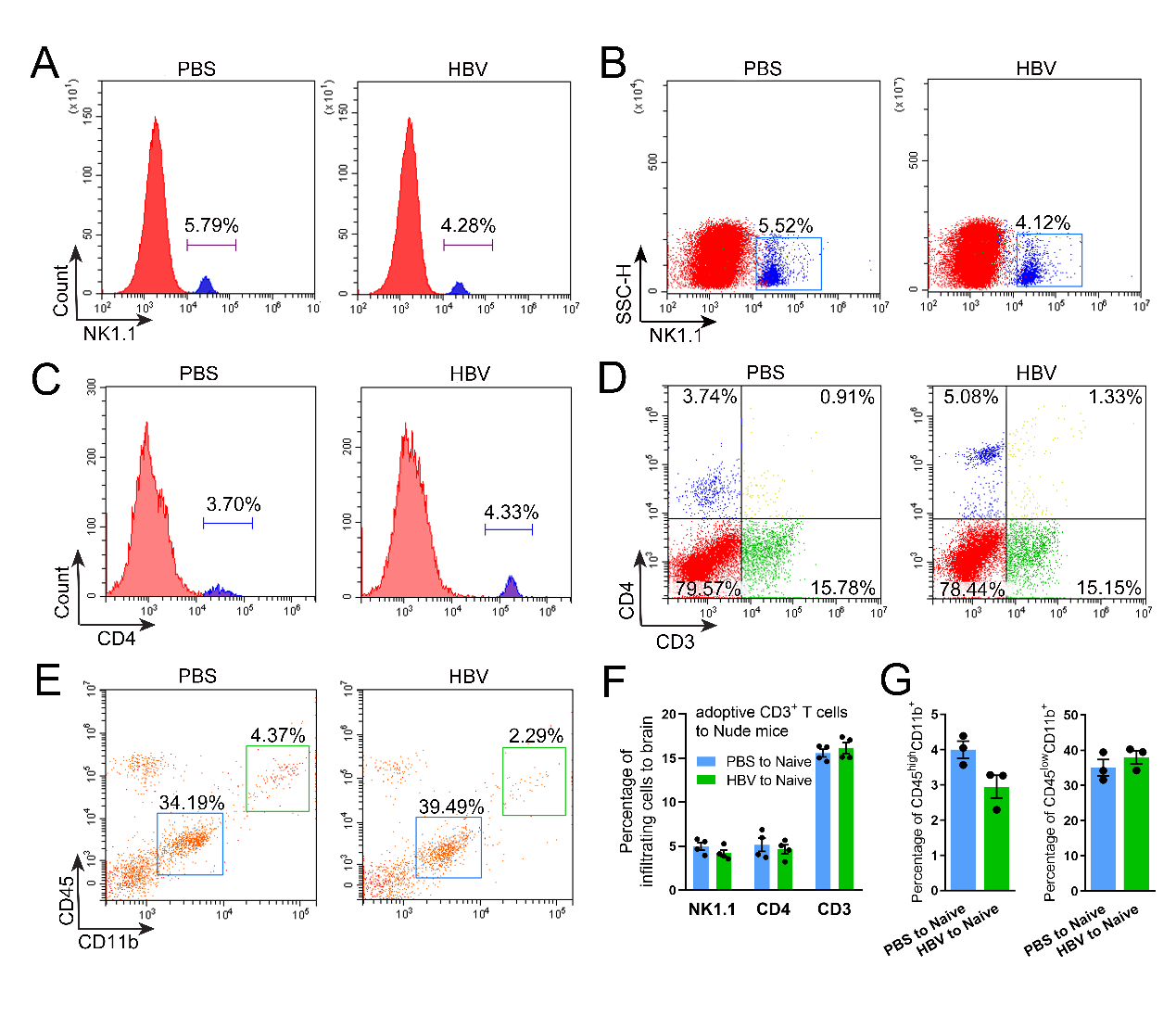


Supplementary Figure 2. Proportions of immune cells in the brain following transfer of CD3^+^ T cells immunized against HBV.

(A-E) Following the adoptive transfer of CD3^+^ T cells from HBV mice or PBS mice (HBV to Naive, PBS to Naive), the representative flow images of NK cells (A-B), CD4^+^ T cells (C-D), macrophages (CD11b^+^/CD45^high^) and microglia (CD11b^+^/CD45^low^) (E) of the total CD45^+^ immune cells. (F-G) Quantitative analysis of the percentage of NK cells, CD4^+^ T cells, macrophages (CD11b^+^/CD45^high^), and microglia (CD11b^+^/CD45^low^) of the total immune cells. Student’s *t*-test (two-tailed), *n* = 5 mice per group. Each dot on the dot plot represents one mouse.


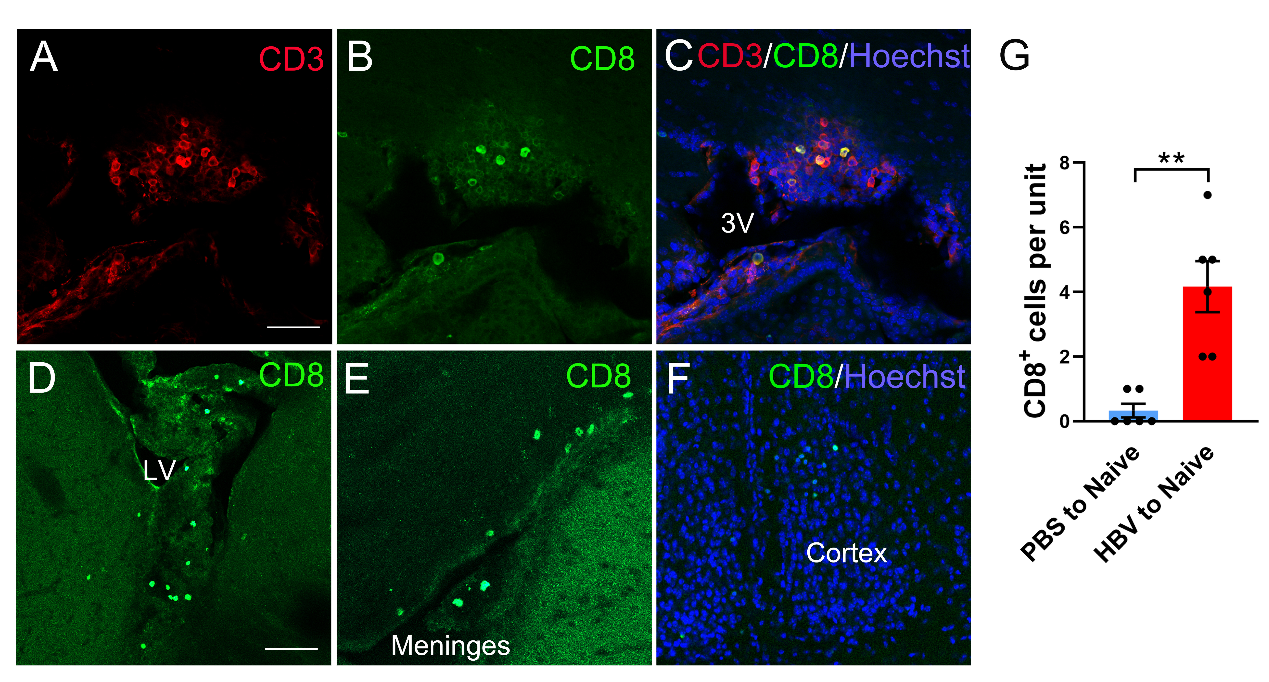


Supplementary Figure 3. CD3^+^ and CD8^+^ T lymphocytes were detected in the brain ventricles and parenchyma in the Naive mice after transferring T cells.

(A-F) Immunostaining with CD3^+^ T cells (red), CD8^+^ T cells (green), and Hoechst (blue) in the 3^rd^ ventricle. (A-C) lateral ventricle (D), meninges (E), parenchyma around the meninges (F), and cortex. (G) Quantitative immunofluorescence analysis of CD8-labeled cells in the ventricles of each group. 3V: third ventricle; LV: lateral ventricle. Scale bar: 100 μm. (G) Mann-Whitney U-test (two-tailed) of CD8**^+^** T cells from brain parenchyma, *n* = 6 mice, ***P* < 0.01. Each dot on the dot plot represents one mouse.


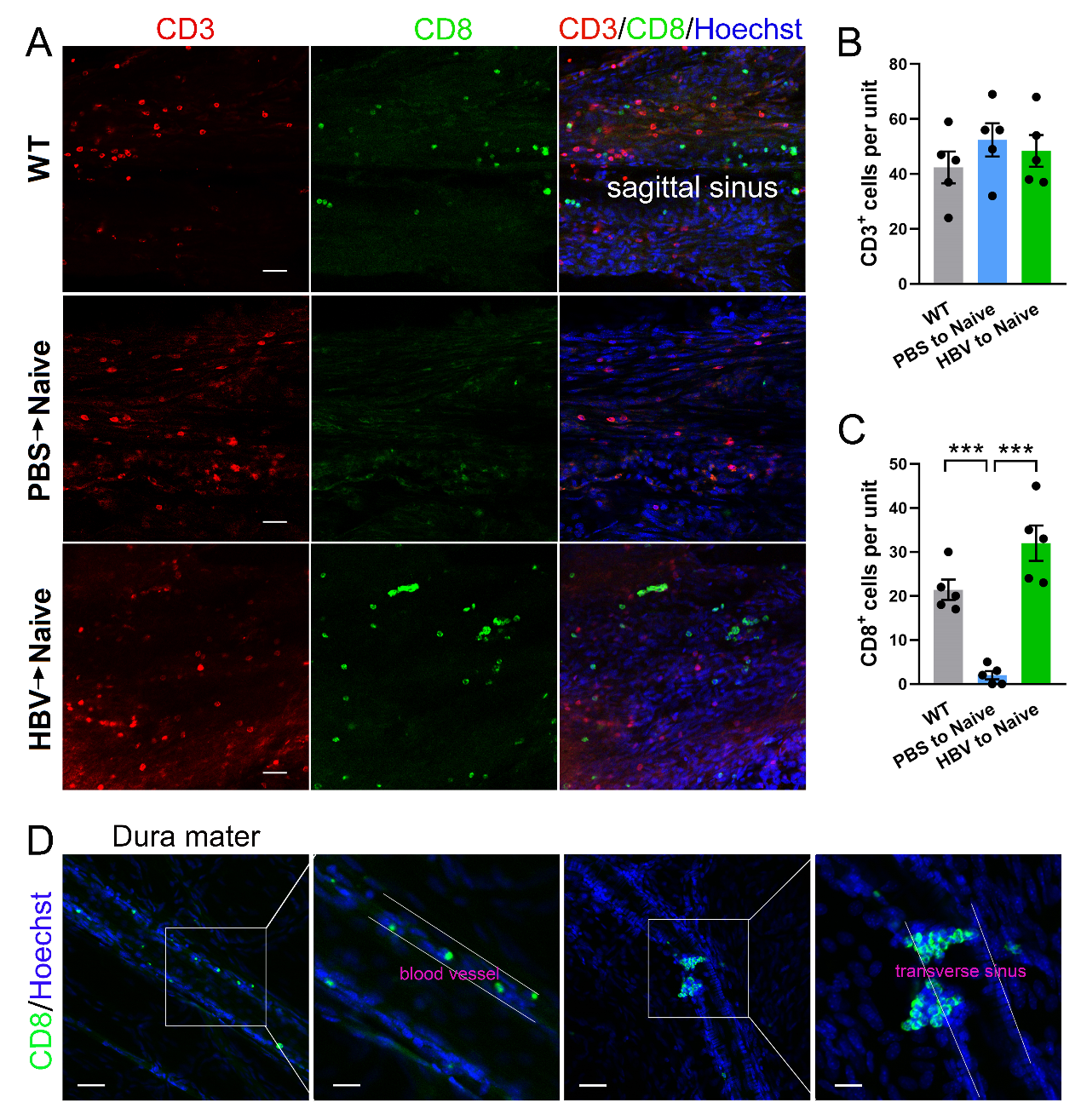


Supplementary Figure 4. Recruitment of CD8^+^ T cells to dura mater from the periphery.

(A) Representative confocal images of the dura mater stained for CD3^+^ T cells (red), CD8^+^ T cells (green), and Hoechst^+^ nuclei (blue) in the sagittal sinus from wide-type (WT) mice, PBS-T cells to Naive mice (PBS to Naive), and HBV-T cells to Naive mice (HBV to Naive). (B-C) Quantitative analysis of CD3^+^ T cell and CD8^+^ T cell counts in the dura mater from three groups. (D)The representative images staining of CD8^+^ T cells passing through or accumulated in blood vessel (left white box) and transverse sinus (right white box) in dura mater from HBV to Naive mice. Scale bar: 50 μm in A, (B-C）one-way ANOVA, *n* = 5 mice per group, ****P* < 0.001. Each dot on the dot plot represents one mouse.


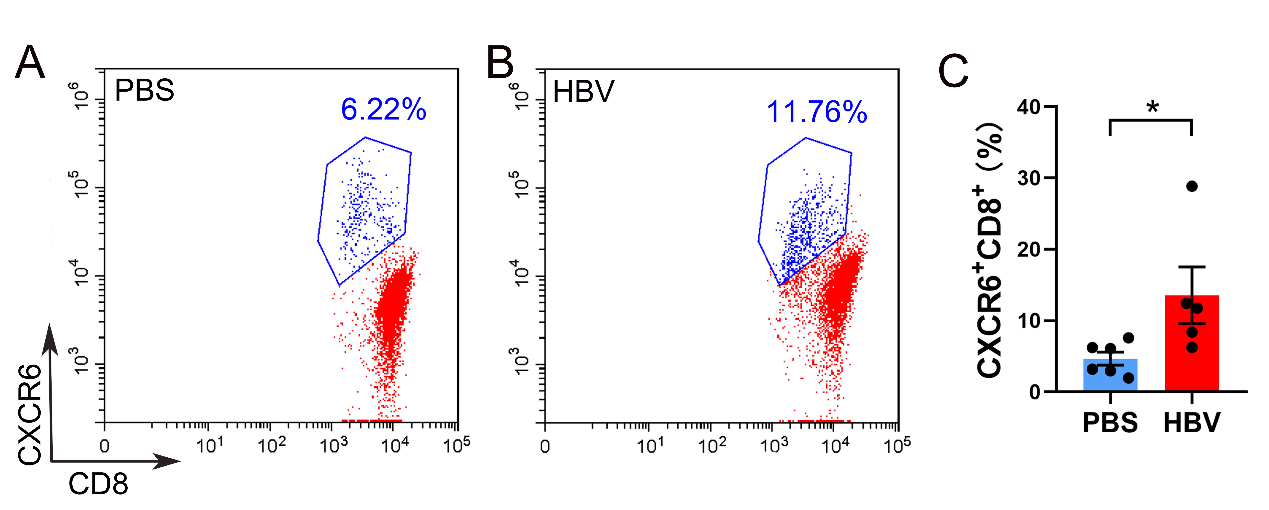


Supplementary Figure 5. CXCR6^+^CD8^+^T cells in the spleen were increased in HBV-treated mice

(A-C) Quantitative analysis of the percentage of CD8^+^ T cells expressing CXCR6 in CD8^+^ T cells in HBV mice and PBS mice by flow cytometry, Student’s *t-*test (two-tailed), *n*=5-6 mice per group, **P* < 0.05. Each dot on the dot plot represents one mouse.


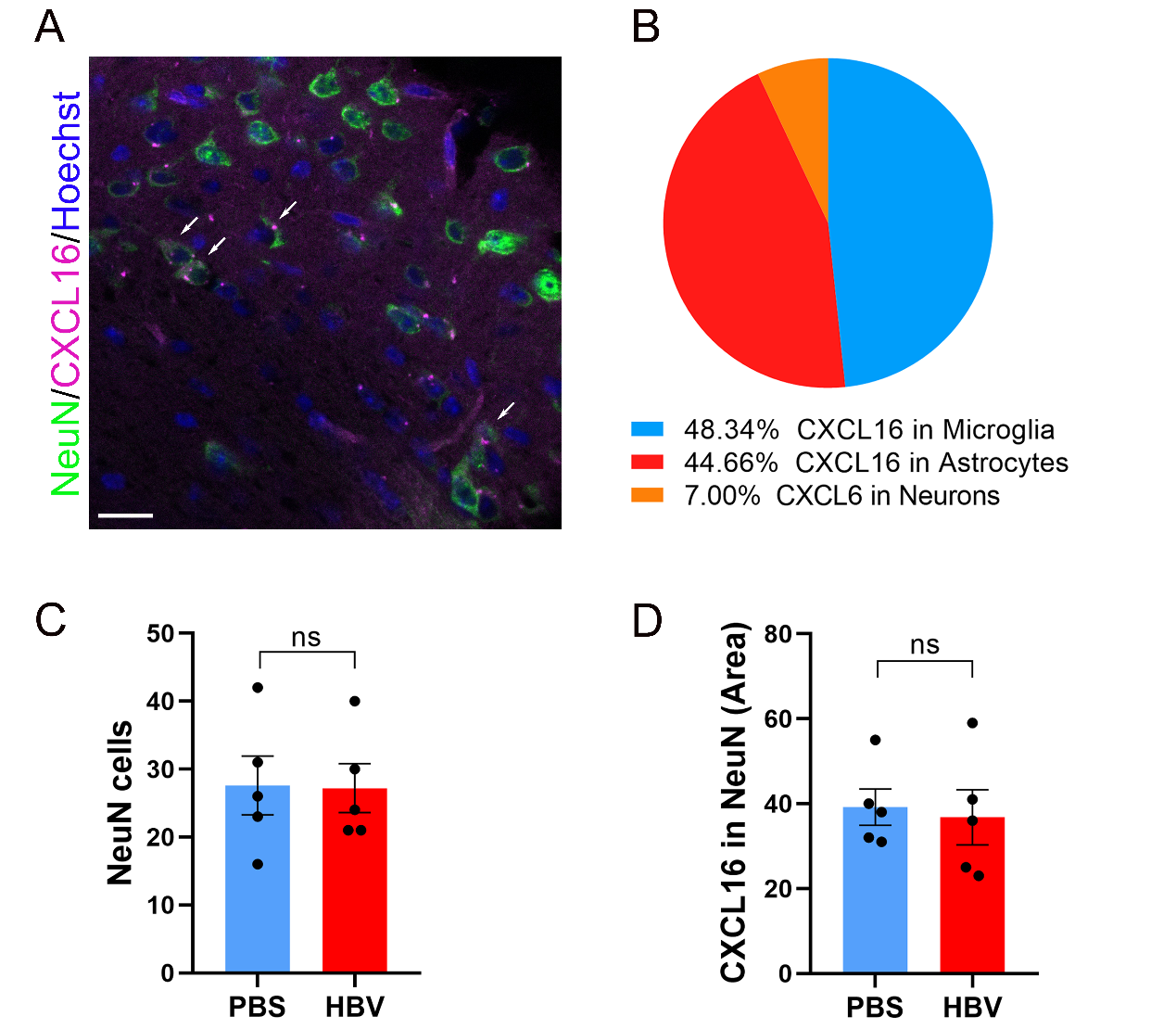


Supplementary Figure 6. CXCL16 expression in neurons and glial cells in the brain

(A) Representative confocal images of NeuN^+^ neurons (green), CXCL16 staining (magenta, indicated by white arrow) and Hoechst (blue) in HBV-treated mice. (B) The percentage of CXCL16 expression in microglia (Iba-1^+^), astrocytes (GFAP^+^), and Neurons (NeuN^+^). (C-D) Quantitative analysis of the number of neurons and CXCL16 expression in neurons in HBV mice and PBS mice. Scale bar: 50 μm. (C-D) Student's *t*-test (two-tailed), ns: non-significant; *n*=5 mice per group. Each dot on the dot plot represents one mouse.

Supplementary Table 1 CXCL16 area in brain cells

|  | Groups | |
| --- | --- | --- |
| Brain cells | PBS | HBV |
| Microglia | 60 | 402 |
|  | 94 | 164 |
|  | 136 | 742 |
|  | 122 | 175 |
|  | 177 | 285 |
|  | 109 | 411 |
|  | 196 | 179 |
| Astrocytes |  |  |
|  | 144 | 410 |
|  | 95 | 456 |
|  | 113 | 426 |
|  | 319 | 423 |
|  | 190 | 349 |
| Neurons |  |  |
|  | 31 | 59 |
|  | 55 | 41 |
|  | 40 | 36 |
|  | 32 | 23 |
|  | 38 | 25 |
| CXCL16 in Microglia/% | 45.82% | 44.13% |
| CXCL16 in Astrocytes/% | 51.94% | 44.81% |
| CXCL16 in Neurons/% | 10.05% | 3.99% |
